# *Arabidopsis* DELLA Protein Degradation Is Controlled by a Type-One Protein Phosphatase, TOPP4

**DOI:** 10.1371/journal.pgen.1004464

**Published:** 2014-07-10

**Authors:** Qianqian Qin, Wei Wang, Xiaola Guo, Jing Yue, Yan Huang, Xiufei Xu, Jia Li, Suiwen Hou

**Affiliations:** Ministry of Education Key Laboratory of Cell Activities and Stress Adaptations, School of Life Sciences, Lanzhou University, Lanzhou, People's Republic of China; Peking University, China

## Abstract

Gibberellins (GAs) are a class of important phytohormones regulating a variety of physiological processes during normal plant growth and development. One of the major events during GA-mediated growth is the degradation of DELLA proteins, key negative regulators of GA signaling pathway. The stability of DELLA proteins is thought to be controlled by protein phosphorylation and dephosphorylation. Up to date, no phosphatase involved in this process has been identified. We have identified a dwarfed dominant-negative *Arabidopsis* mutant, named *topp4-1*. Reduced expression of *TOPP4* using an artificial microRNA strategy also resulted in a dwarfed phenotype. Genetic and biochemical analyses indicated that TOPP4 regulates GA signal transduction mainly via promoting DELLA protein degradation. The severely dwarfed *topp4-1* phenotypes were partially rescued by the DELLA deficient mutants *rga-t2* and *gai-t6*, suggesting that the DELLA proteins RGA and GAI are required for the biological function of TOPP4. Both RGA and GAI were greatly accumulated in *topp4-1* but significantly decreased in *35S-TOPP4* transgenic plants compared to wild-type plants. Further analyses demonstrated that TOPP4 is able to directly bind and dephosphorylate RGA and GAI, confirming that the TOPP4-controlled phosphorylation status of DELLAs is associated with their stability. These studies provide direct evidence for a crucial role of protein dephosphorylation mediated by TOPP4 in the GA signaling pathway.

## Author Summary

Gibberellins (GAs) are essential regulators of plant growth and development. They are tightly related to crop productivity in the first “green revolution.” GA triggers its responses by targeting DELLA proteins, the important repressors, for degradation. This process is believed to be regulated by protein phosphorylation and dephosphorylation, but there are not any reports describing the identification of phosphatases regulating this critical event. By screening an ethyl methane sulfonate (EMS)-mutagenized *Arabidopsis thaliana* population, we identified a protein phosphatase TOPP4, a member of protein phosphatase 1 (PP1), that acts as a positive regulator in the GA signaling pathway. TOPP4 promotes the GA-induced degradation of DELLA proteins by directly dephosphorylating these proteins. This study provides an important insight for the switch role of protein phosphorylation and dephosphorylation in GA signal transduction and sheds light on PP1 protein phosphatases in regulating plant growth and development.

## Introduction

Gibberellins (GAs) are a class of major plant hormones mediating almost all physiological events during normal plant lifespan, including seed germination, leaf formation, cell elongation and flowering time control, etc [1]–[3]. In recent decades, several molecular components essential for GA signal transduction have been characterized using genetic and biochemical approaches [4]. One group of these components is nuclear-localized DELLA proteins. These proteins belong to a subset of GRAS family of putative transcriptional regulators that contain specific DELLA motifs at their N-termini and conserved GRAS domains at their C-termini. They are key repressors of the GA signaling pathway [2], [5]. In *Arabidopsis* genome, there are five DELLA proteins, designated as GA INSENSITIVE (GAI), REPRESSOR OF *ga1-3* (RGA), REPRESSOR OF *ga1-3*-LIKE protein (RGL)1, RGL2, and RGL3, respectively [6]–[9]. Genetic analyses indicated that these DELLAs have overlapping and sometimes distinctive roles in regulating plant growth and development. For example, GAI and RGA are important for stem elongation [10], [11]; RGL2 regulates seed germination [9]; whereas RGA, RGL1, and RGL2 are involved in floral development [12]–[14]. A major GA signaling cascade has recently been elucidated. In the nucleus, GA is perceived by its receptor, GIBBERELLIN INSENSITIVE DWARF 1 (GID1) [15], [16]. The formation of the ligand-receptor complex enhances the interaction of GID1 with the DELLA domain of DELLA proteins [17], leading either to their direct inactivation [18] or ubiquitination by SCF^SLY1/GID2^ (Skp1-Cullin-F-box protein complex) E3 ligase [19]–[22]. Ubiquitinated DELLA proteins are subsequently degraded by the 26S proteasome system, triggering GA responses.

In the absence of GA, on the other hand, DELLAs are stably localized in the nucleus where they interact with other transcription factors to inhibit the transcription of GA-responsive genes [23]–[27], restraining growth and development processes in *Arabidopsis* [6], [9], [11], [13]. DELLAs also promote the transcription of *GID1b* by interacting with other transcription factors, and maintain GA homeostasis by up-regulating the expression of GA biosynthetic genes *GA 20-oxidases 2* (*GA20ox2*) and *GA 3-oxidases 1* (*GA3ox1*) [28]. Moreover, DELLAs are important integrators of other phytohormones, including auxin, ethylene, abscisic acid (ABA), brassinosteroid (BR), and jasmonate (JA) [29]–[31], and environmental factors, such as light [24], [25], cold [32], and salt [33]. Very recently, these proteins were found to regulate cortical microtubule organization [34]. Besides ubiquitination and glycosylation [19], [22], [35], limited evidence also suggested that DELLA proteins are regulated by reversible protein phosphorylation and dephosphorylation [36], [37]. The detailed molecular mechanisms, however, are poorly understood and the protein phosphatases involved in this process have not been reported.

Protein phosphatases 1 (PP1s) are a major group of serine/threonine (Ser/Thr) protein phosphatases. They are expressed ubiquitously in eukaryotes [38], regulating diverse cellular processes in animals [39], although their functions in plants are uncertain. In *Arabidopsis*, PP1s are referred to as type-one protein phosphatases (TOPPs) [40]. Previous studies indicated that they regulate embryonic development and blue light-dependent stomatal opening [41], [42]. In general, molecular mechanisms of TOPPs in regulating plant growth and development are not well studied.

Using a forward genetic approach, we identified that one of the nine TOPPs in *Arabidopsis*, TOPP4, is involved in GA signal transduction. Biochemical analyses revealed that TOPP4 directly interacts with and dephosphorylates DELLA proteins RGA and GAI, promoting the GA-induced destabilization of these two proteins. A novel regulatory mechanism for protein dephosphorylation in the GA signaling pathway via TOPP4 is proposed.

## Results

### *topp4-1* Was Identified from EMS-Mutagenized *Arabidopsis* Pool

**Figure 1:**
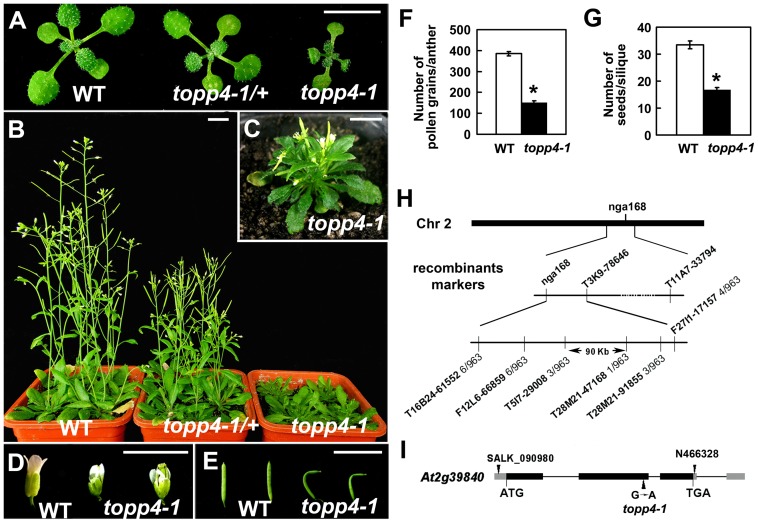
Extremely dwarfed phenotypes of the *topp4-1* mutant and map-based cloning strategy of the *topp4-1* locus. (**A**) Representative 2-week-old wild-type, heterozygous, and homozygous mutant seedlings. (**B**) Representative 6-week-old plants. (**C**) A 6-week-old *topp4-1* homozygous plant. (**D**) Flowers from *topp4-1* mutant are small with irregular and narrow sepals. (**E**) Siliques from *topp4-1* mutant are twisted with few seeds. (**F**) Numbers of pollen grains per mature anther are reduced in *topp4-1* homozygous plants. (**G**) Numbers of seeds per mature silique are reduced in *topp4-1* homozygous plants. (**H**) *topp4-1* was mapped on chromosome 2. Numbers of recombinants are shown below the markers. (**I**) Genomic structure of *TOPP4*. Thick black boxes represent exons and lines between the boxes represent introns. Gray boxes represent untranslated regions. Arrowheads represent three *TOPP4* alleles identified through this research (*topp4-1*) or obtained from other resources (N466328 and SALK_090980). Scale bars = 1 cm. Asterisks in (**F**) and (**G**) represent statistic differences based on Student's *t* test with P<0.05. Error bars in (**F**) and (**G**) represent standard error (SE) (n = 30).

We isolated an extremely dwarfed mutant from a 2000 M_2_ ethyl methane sulfonate (EMS)-mutagenized *Arabidopsis* population. The dwarfed plant was back-crossed three times with wild-type Col-0 and the resulting mutant was used in all studies presented. The mutant exhibits lack of apical dominance and aberrant leaf phyllotaxy (Figure 1A–C). Compared to wild-type plants, the mutant has tiny, curled, and dark-green rosette leaves (Figure 1A–C), delayed flowering (Figure S1), smaller flowers with irregular and narrow sepals (Figure 1D), partially twisted petals and siliques (Figure 1D–E), reduced mature pollen grains in anthers (Figure 1F), and fewer seeds in mature siliques (Figure 1G). This mutant resembles GA deficient or signaling mutants, since the dwarfism, reduced rosette radius, delayed flowering time, and high chlorophyll content of the mutant are similar to those of *ga1-3*, *ga4*, *gai-1*, and *gid1a/b/c* [16], [43], [44] (Figure S1). The mature heterozygous mutant plants were semi-dwarfed with clustered siliques and no apical dominance, suggesting that the traits were inherited in a semi-dominant manner (Figure 1B). When the mutant was back-crossed to Col-0, the F_2_ population resulting from self pollination had a segregation ratio of 97∶257∶118 (normal plants∶semi-dwarf plants∶dwarf plants), close to the expected 1∶2∶1 segregation ratio for a semi-dominant single locus.

Map-based cloning was employed to identify the gene responsible for the mutant phenotype (Figure 1H). The mutant in Col-0 ecotype background was crossed to Landsberg *erecta*-0 (L*er*-0). The extremely dwarfed homozygous plants were selected from the F_2_ population for mapping. The locus was roughly mapped to a site close to a known marker nga168 on chromosome 2. Eight newly developed insertion/deletion (In/Del) and cleaved amplified polymorphic sequence (CAPS) markers were then used for fine mapping (Table S1). The corresponding locus was eventually mapped to a 90-Kb region between markers T5I7-29008 and T28M21-47168, with three and two recombinants for each marker, respectively, in the population of about 1000 individuals. This 90-Kb region contains 32 gene loci according to the gene annotation data obtained from the *Arabidopsis* genome database. We sequenced all 32 genes, and found a G to A single-nucleotide substitution in *At2g39840* that resulted in the conversion of threonine (Thr) to methionine (Met) in amino acid 246 which is near the C terminus of the predicted protein sequence (Figure 1I). *At2g39840* encodes a protein previously named TOPP4 [40]. The mutant was therefore designated as *topp4-1*. The single nucleotide substitution of *topp4-1* did not influence *TOPP4* gene transcription and its protein level (Figure S2).

### Mutated topp4-1 Protein Has a Dominant-Negative Effect on Plant Development

**Figure 2:**
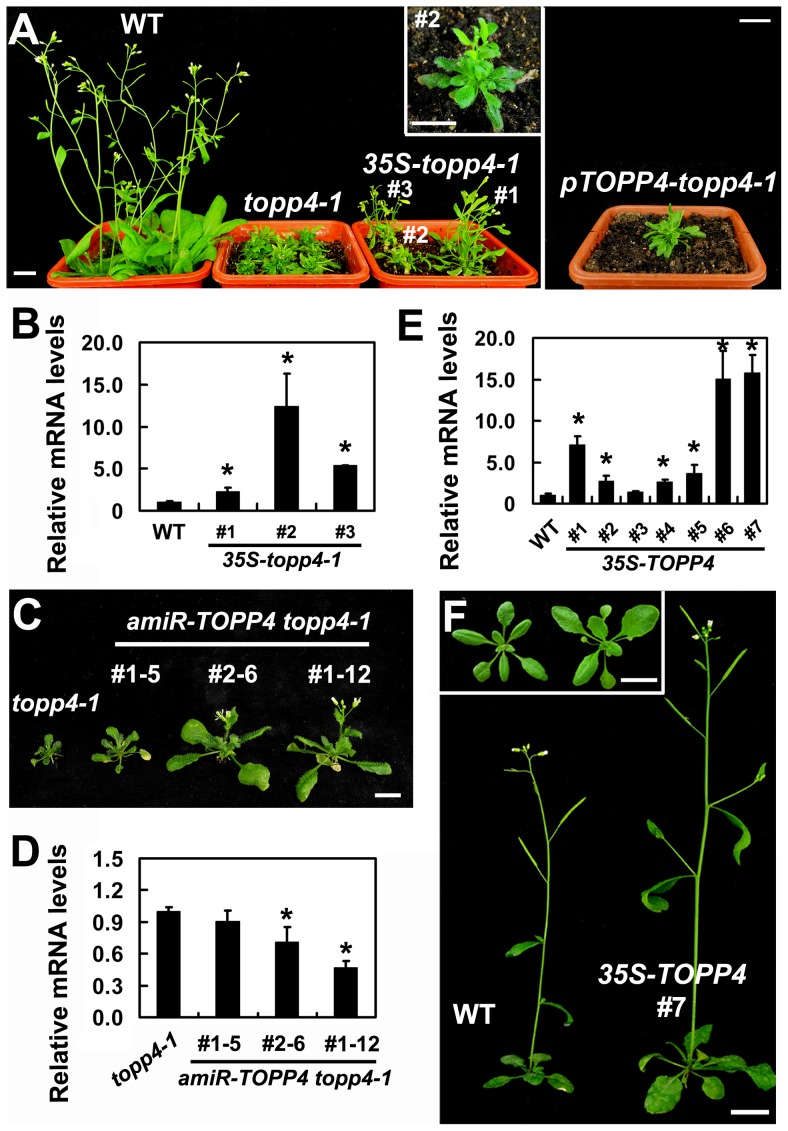
Expression of *35S-topp4-1*, *pTOPP4-topp4-1*, or *35S-TOPP4* in wild-type plants. (**A**) Expression of *35S-topp4-1* or *pTOPP4-topp4-1* in wild type recapitulates the dwarfed phenotypes of *topp4-1*. Five-week-old *35S-topp4-1* transgenic plants are shown on the left, and upper right corner is the higher magnitude picture of the #2 transgenic plant. Five-week-old *pTOPP4-topp4-1* transgenic plant is shown on the right. (**B**) qRT-PCR was used to detect relative expression levels of the *topp4-1* in three *35S-topp4-1* transgenic lines shown in (**A**). The expression level of *TOPP4* in wild type was set to 1.0. (**C**) Representative 4-week-old *amiR-TOPP4 topp4-1* transgenic lines. The first number 1 or 2 of the lines represents transgenic plants generated by *amiR-TOPP4*-1 *or amiR-TOPP4*-2, respectively. (**D**) qRT-PCR was used to detect the relative expression levels of *topp4-1* in three *amiR-TOPP4 topp4-1* transgenic lines shown in (**C**). The expression level of *topp4-1* in the *topp4-1* mutant was set to 1.0. (**E**) qRT-PCR was used to detect the relative expression levels of *TOPP4* in seven 2-week-old transgenic seedlings overexpressing *TOPP4*. The expression level in wild type was set to 1.0. (**F**) Five-week-old transgenic plant overexpressing *TOPP4* shows increased inflorescence and enlarged organ size compared to the wild-type plant. One representative line (#7) is shown. Three-week-old wild-type plant (left) and *35S-TOPP4* #7 (right) are shown in the upper left corner. Scale bars = 1 cm in (**A**) and (**F**), 0.5 cm in (**C**). Asterisks in (**B**), (**D**) and (**E**) represent statistic differences based on Student's *t* test with P<0.05. In (**B**), (**D**) and (**E**), error bars represent SE (n = 3).

Our genetic result suggested that topp4-1 protein should have a dominant-negative effect. To confirm that, we made transgenic plants by introducing a construct containing the full-length cDNA of *topp4-1* driven by a cauliflower mosaic virus (CaMV) 35S promoter (*35S-topp4-1*) into wild-type plants. More than 20 independent transgenic lines were obtained. All of them showed dwarfed phenotypes similar to those of the *topp4-1* mutant plants, such as lacking apical dominance, abnormal leaf phyllotaxy and curled rosette leaves, and reduced sterility (Figure 2A). The severity of the defective phenotypes appeared to be positively correlated with the expression levels of the *topp4-1* gene (Figure 2A–B). Most of the transgenic seedlings died before flowering. We also generated a construct containing the cDNA of *topp4-1* driven by its own promoter (*pTOPP4-topp4-1*) and transformed it into wild-type plants. The *pTOPP4-topp4-1* transgenic plants showed *topp4-1* mutant-like phenotypes (Figure 2A). Furthermore, we constructed two different artificial microRNA (amiRNA) vectors that specifically target the *TOPP4/topp4-1* gene (*amiR-TOPP4*-1 and *amiR-TOPP4*-2) [45]. The *topp4-1* mutant could be partially rescued regarding to inflorescence height, rosette leaves, and flowering time when the mutated *topp4-1* gene was knocked down by amiRNA (Figure 2C). The recovery effect was positively correlated with the knocked down level of the *topp4-1* gene (Figure 2D). These results clearly indicated that the mutated topp4-1 protein caused a dominant-negative effect on plant growth.

### Overexpression of *TOPP4* Rescued *topp4-1*, and *amiR-TOPP4* Transgenic Lines Also Showed Dwarfed Phenotypes

**Figure 3:**
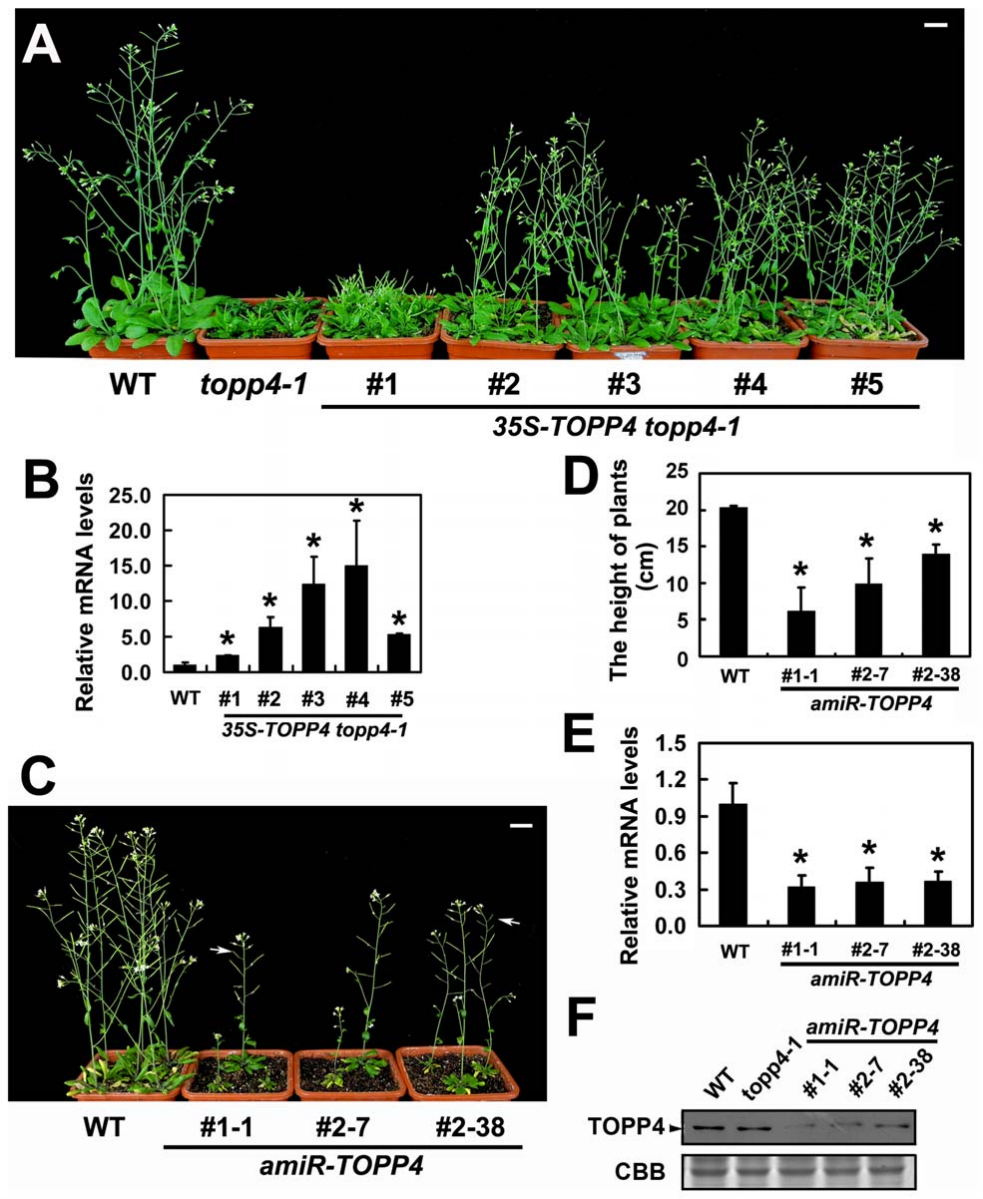
Overexpression of *TOPP4* in *topp4-1* rescued the severely dwarfed phenotype, and amiRNA lines of *TOPP4* showed dwarfed phenotypes. (**A**) Representative 6-week-old *35S-TOPP4 topp4-1* transgenic lines. (**B**) qRT-PCR was used to detect the relative expression levels of *TOPP4* in five *35S-TOPP4 topp4-1* transgenic lines shown in (**A**). The expression level of *TOPP4* in wild type was set to 1.0. (**C**) Representative 5-week-old *amiR-TOPP4* transgenic lines. The first number 1 or 2 of the lines represents transgenic plants generated by *amiR-TOPP4*-1 *or amiR-TOPP4*-2, respectively. The white arrows indicate partial-sterile siliques. (**D**) The height of 5-week-old wild type and three *amiR-TOPP4* transgenic lines. (**E**) qRT-PCR was used to detect the relative expression levels of *TOPP4* in three amiRNA lines shown in (**C**). The expression level of *TOPP4* in wild type was set to 1.0. (**F**) The protein levels of TOPP4 in wild type, *topp4-1*, and three amiRNA lines of *TOPP4*. The coomassie brilliant blue-stained RbcS protein was used as loading controls. Scale bars = 1 cm in (**A**) and (**C**). Asterisks in (**B**), (**D**) and (**E**) represent statistic differences based on Student's *t* test with P<0.05. In (**B**), (**D**) and (**E**), error bars represent SE (n = 3).

To confirm whether the single nucleotide substitution of *TOPP4* was responsible for the defective phenotypes, we transformed *topp4-1* plants with a 1.6-Kb *TOPP4* genomic fragment under the control of CaMV 35S promoter (*35S-TOPP4*) using an *Agrobacterium tumefaciens*-mediated floral dipping method [46]. Ten independent T_1_ transgenic lines were obtained, all of which showed semi-dwarfed phenotypes. Five independent lines apparently containing a single insertion were selected for further studies. Approximately 25% of the plants from each of the four lines (#2–#5) had obviously complemented phenotypes in the T_2_ generation: they were significantly taller than the *topp4-1* background but still a little shorter than wild-type plants (Figure 3A). However, one line (#1) had a weak complemented phenotype (Figure 3A). Subsequent quantitative reverse transcription-polymerase chain reaction (qRT-PCR) revealed that this line had relatively low expression level of *TOPP4* compared to the other four lines (Figure 3B). Therefore, it seemed that the inflorescence heights of the transgenic plants were positively correlated with the expression levels of *TOPP4* (Figure 3A–B). The point mutation of *TOPP4* was responsible for the extremely dwarfed phenotype of *topp4-1*. In addition, we transformed *TOPP4* gene under the control of its own promoter (*pTOPP4-TOPP4*) into the *topp4-1* mutant. The T_2_ transgenic lines showed increased rosette width than the *topp4-1* mutant, but the height and the curled leaf phenotypes were not altered (Figure S3).

In order to analyze the phenotype of the loss-of-function mutants, we searched the SALK and GABI-Kat T-DNA insertion databases for T-DNA insertion alleles of *AT2g39840*. Two independent T-DNA lines, SALK_090980 and N466328, were identified by PCR-based analyses (Figure S4A–C). Neither of them had obvious mutant phenotypes. In SALK_090980, the T-DNA is inserted 92 nucleotides upstream of the initiation codon ATG (Figure 1I). This insertion did not alter the transcription level of *TOPP4* (Figure S4D). In N466328, the T-DNA is located in the 3′ untranslated region, 23 bp after the stop codon TGA (Figure 1I). qRT-PCR analysis showed that, the expression level of *TOPP4* is decreased in this mutant (Figure S4D). N466328 did not show obvious phenotype, possibly because it is a knock-down rather than a null mutant. It still expresses about 40% of the wild-type level of *TOPP4*.

To test whether further reducing the expression level of *TOPP4* can finally result in a dwarfed phenotype, we transformed the two *amiR-TOPP4* vectors into wild-type plants. Four plants from 86 T_2_ transgenic lines of *amiR-TOPP4*-1 and five plants from 128 T_2_ transgenic lines of *amiR-TOPP4*-2 exhibited dwarfed phenotypes. Three representative plants, *amiR-TOPP4* #1-1, #2–7 and #2–38, were selected for subsequent analyses. They showed shorter inflorescences, curled leaves, decreased fertility, and retarded growth (Figures 3C–D and S5). Compared to wild type, the expression level of the *TOPP4* gene in these amiRNA transgenic lines was decreased to about 30% of their wild type counterpart (Figure 3E). Correspondingly, TOPP4 protein also dramatically declined in these lines (Figure 3F).

### Overexpression of *TOPP4* in the Wild Type Resulted in Enlarged Organs

To investigate the effect of TOPP4 on plant growth and development, we transformed wild-type plants with the *35S-TOPP4* construct. More than 20 independent lines were obtained. The constitutive expression of *TOPP4* in these lines was confirmed by qRT-PCR (Figure 2E). Interestingly, all of them had enlarged organs compared to wild-type plants (Figure S6A). One of the representative transgenic lines (#7) with the highest expression level of *TOPP4* was selected for subsequent analyses (Figure 2F). Overexpression of *TOPP4* in wild-type plants resulted in elongated hypocotyls, increased plant height, thickened stems, and enlarged rosette leaves, inflorescences, flowers and siliques (Figures 2F and S6B–H).

### *TOPP4* Is Ubiquitously Expressed and TOPP4 Protein Is Mainly Localized in the Nucleus and at the Plasma Membrane

**Figure 4:**
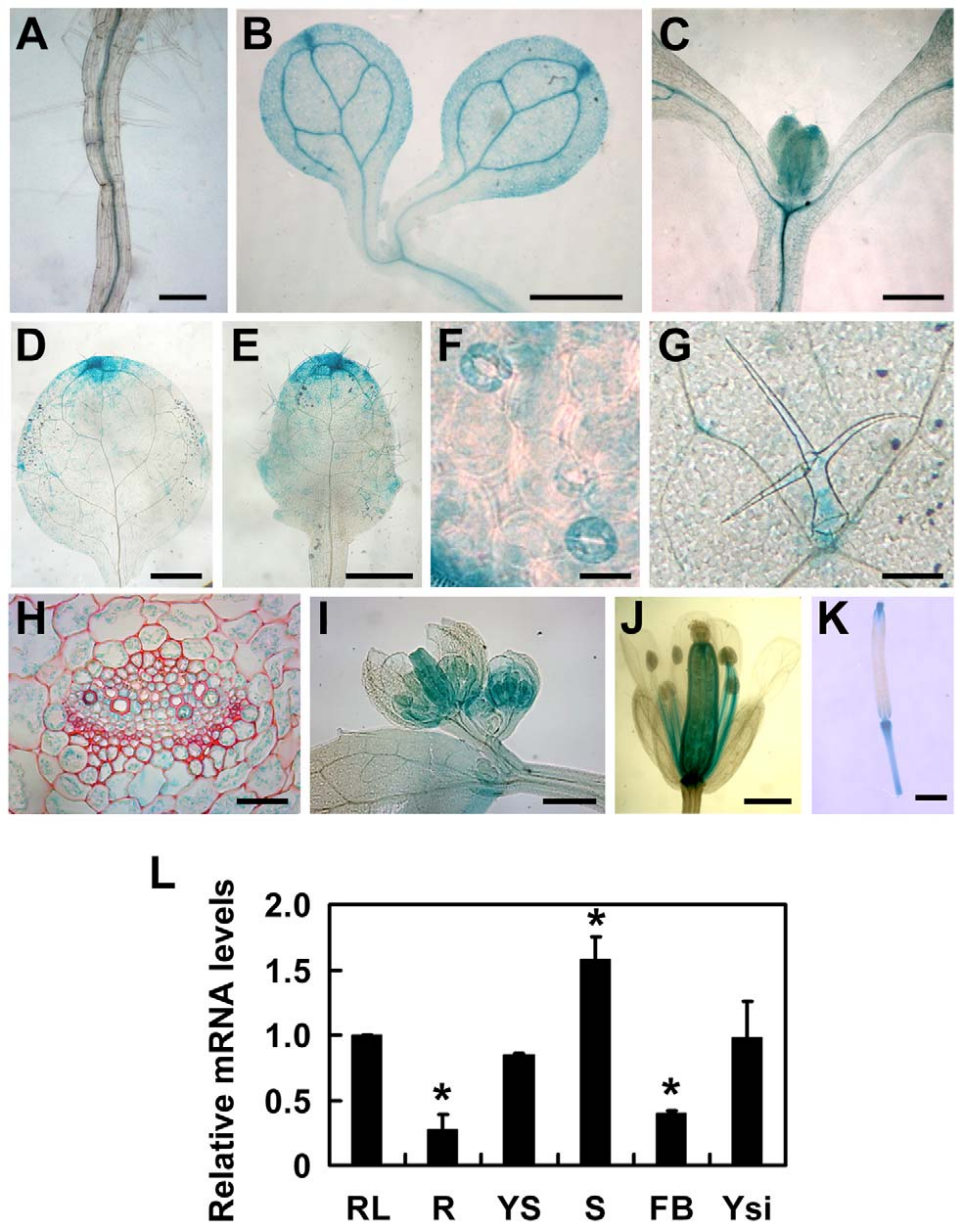
Tissue-specific expression patterns of the *TOPP4* gene. (**A**)–(**K**) GUS activity was detected in various organs of *pTOPP4-GUS* transgenic plants at different development stages. (**A**) Root from a 7-day-old transgenic plant. (**B**) Cotyledons of a 7-day-old seedling. (**C**) Shoot apical meristem from a 12-day-old seedling. (**D**) One of the first pair of true leaves from a 3-week-old seedling. (**E**) One of the second pair of true leaves from a 3-week-old seedling. (**F**) Stomata from a mature rosette leaf. (**G**) Trichome from a mature rosette leaf. (**H**) GUS and safranin-stained 7-µm cross section of a mature rosette leaf. (**I**) Young flowers from a 4-week-old plant. (**J**) Mature flower from a 4-week-old plant. (**K**) Silique from a 4-week-old plant. (**L**) qRT-PCR was used to detect the relative expression levels of *TOPP4* gene in different organs. Rosette leaves (RL), roots (R), young seedlings (YS), stems (S), flower buds (FB), young siliques (Ysi). The expression level of *TOPP4* in rosette leaves was set to 1.0. Asterisks represent statistic differences based on Student's *t* test with P<0.05. Error bars represent SE (n = 3). Scale bars = 100 µm in (**A**); 1 mm in (**B**)–(**E**), and (**K**); 20 µm in (**F**) and (**H**); 200 µm in (**G**), (**I**), and (**J**).

To understand the expression patterns of *TOPP4*, a 2-Kb fragment upstream of the translation initiation codon ATG of the *TOPP4* gene was fused to the β-glucuronidase (GUS) reporter gene in the binary vector pCAMBIA 1300-GUS (*pTOPP4-GUS*) and this vector was transformed into wild-type plants. More than 20 transgenic lines were obtained, and T_2_ generation transgenic plants were used for GUS staining analyses. Because all transgenic lines showed consistent expression patterns, only one representative line, *pTOPP4-GUS* #8, was used for further analyses. In young seedlings, GUS staining was detected in the stele of roots and hypocotyls (Figure 4A–C), the vascular bundles of cotyledons (Figure 4B), and newly emerging leaves (Figure 4C). In mature leaves of 3-week-old plants, GUS expression was observed mainly in tips, blades (Figure 4D–E), stomata (Figure 4F), and the base of trichomes (Figure 4G). Cross sections of the rosette leaves also revealed GUS activity in vascular bundles and mesophyll cells (Figure 4H). Furthermore, GUS staining was observed in pistil and stamen filaments of flowers (Figure 4I–J), as well as the apex and the base of elongating siliques (Figure 4K). In conclusion, *TOPP4* is ubiquitously expressed in various organs throughout development, suggesting its diverse and crucial functions in plant developmental processes. qRT-PCR analyses were consistent with the GUS staining results and showed relatively higher expression levels of *TOPP4* in stems, rosette leaves, and young siliques (Figure 4L).

**Figure 5:**
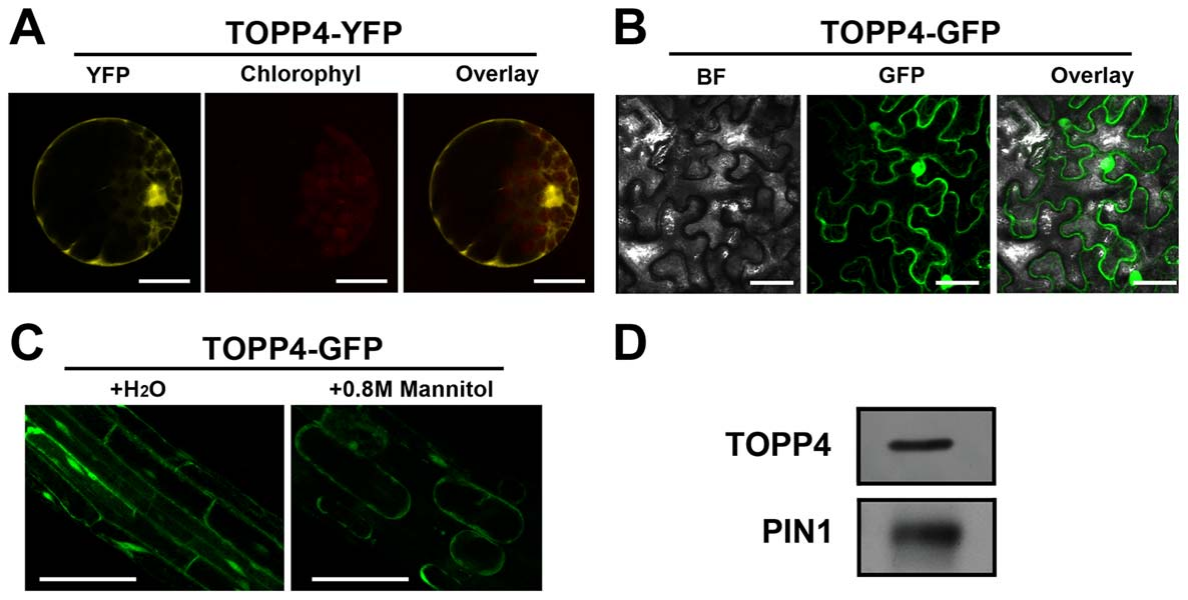
Subcellular localization of the TOPP4 protein. (**A**) Subcellular localization of TOPP4-YFP in *Arabidopsis* protoplasts indicated that TOPP4 is mainly localized in the nucleus and at the plasma membrane. (**B**) TOPP4-GFP transiently expressed in pavement cells of a tobacco leaf. BF, bright field; GFP, GFP fluorescence; overlay, merged image. (**C**) Plasmolysis studies showed that TOPP4-GFP is located on plasma membrane in root cells from 10-day-old *35S-TOPP4-GFP* transgenic plants. (**D**) Immunoblotting detection of the TOPP4 protein in purified plasma membrane fraction of wild-type seedlings. PIN1 protein was used as a positive control. Anti-GFP and anti-PIN1 antibodies were used for detecting TOPP4 and PIN1, respectively. Scale bars = 20 µm in (**A**); 40 µm in (**B**) and (**C**).

To reveal subcellular localization of the TOPP4 protein, a yellow fluorescent protein (YFP)-tagged TOPP4 was transiently expressed in mesophyll protoplasts from wild-type plants. TOPP4-YFP protein was ubiquitously distributed in cells. It was mainly localized in the nucleus and at the plasma membrane (Figure 5A). TOPP4-YFP signals were also found in cytoplasm (Figure 5A). Analyses of the transient expression pattern of a green fluorescent protein (GFP)-tagged TOPP4 in *Nicotiana benthamiana* leaves confirmed the subcellular distribution of the TOPP4 protein (Figure 5B). The localization of TOPP4 to the plasma membrane was also verified by plasmolyzing roots of 10-day-old *35S-TOPP4-GFP* plants with 0.8 M mannitol for 1 h. Confocal analysis of these roots revealed that TOPP4-GFP was associated with the plasma membrane (Figure 5C). This result was further confirmed by an immunoblotting assay using purified plasma membrane fraction (Figure 5D).

### *TOPP4* Is Involved in GA Signal Transduction and GA Regulates the Level of TOPP4 Protein

**Figure 6:**
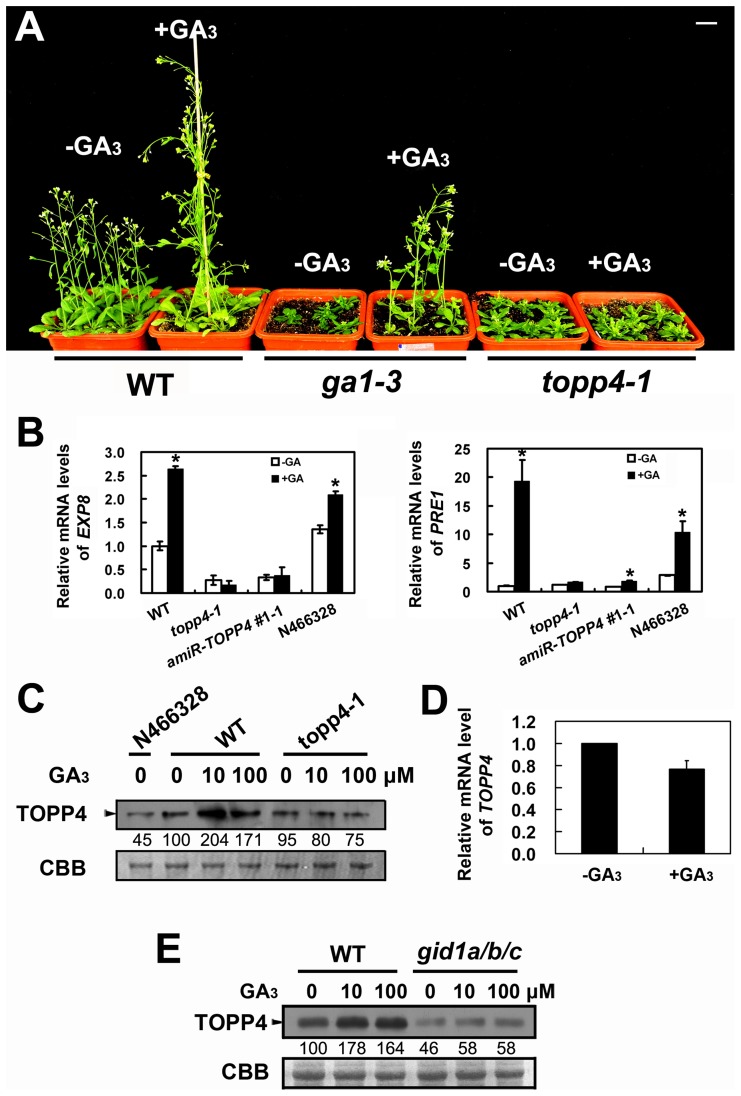
*topp4-1* is insensitive to exogenously applied GA_3_, and GA enhances the TOPP4 protein level through a GA-GID1 pathway. (**A**) Four-week-old wild-type, *ga1-3*, and *topp4-1* seedlings sprayed with or without 100 µM GA_3_. Scale bar = 1 cm. (**B**) Analysis the expression of GA responsive genes *EXP8* and *PRE1* in wild type, *topp4-1*, *amiR-TOPP4* #1-1, and N466328 by qRT-PCR. The expression levels in wild type were set to 1.0. Asterisks represent statistic differences based on Student's *t* test with P<0.05. Error bars represent SE (n = 3). (**C**) The protein levels of TOPP4 in 2-week-old N466328, and 2-week-old wild-type and *topp4-1* seedlings treated with different concentrations of GA_3_. (**D**) Analysis of the *TOPP4* expression in 2-week-old wild type treated with GA_3_ by qRT-PCR. The expression level in wild type was set to 1.0. Error bar represents SE (n = 3). (**E**) The protein levels of TOPP4 in wild-type and *gid1a/b/c* seedlings treated with different concentrations of GA_3_. Numbers under lanes in (**C**) and (**E**) indicate relative band intensities that were quantified and normalized for each panel. The coomassie brilliant blue-stained RbcS protein was used as loading controls.

The morphological similarity of *topp4-1* plants to GA deficiency or signaling mutants prompted us to examine whether the GA signal transduction is altered in *topp4-1*. Therefore, responses of the mutant to exogenously applied GA_3_ were analyzed. Although GA_3_ rescued the dwarfed phenotype of *ga1-3* as previously reported [47], it did not affect the stem elongation of *topp4-1* (Figure 6A). Moreover, GA_3_ increased the transcription level of GA responsive genes *EXPANSIN A8* (*EXP8*) and *PACLUBUTRAZOL RESISTANCE 1* (*PRE1*) in wild type and N466328 backgrounds, but almost had no effect on these genes in *topp4-1* and *amiR-TOPP4* #1-1 (Figure 6B). These results indicated that *topp4-1* is insensitive to GA and that the GA signaling pathway is blocked in the mutant. Thus, TOPP4 is likely involved in GA signal transduction.

The level of TOPP4 protein after GA_3_ treatment was also examined by immunoblotting using an anti-TOPP4 antibody. A band with a molecular mass between 37 and 55KD was detected, and it was weak in N466328 (Figure 6C), demonstrating that it is the corresponding band of TOPP4 and the anti-TOPP4 antibody is specific. After exogenous GA_3_ treatment, TOPP4 protein was increased in wild-type plants but was almost unaltered in *topp4-1* (Figures 6C and S7). This result suggested that GA may promote TOPP4 protein accumulation in wild-type plants, and this effect is apparently disturbed in *topp4-1*. This might be one of the reasons that *topp4-1* is insensitive to GA. However, the GA_3_-induced TOPP4 protein accumulation was not caused by the increased transcription level of *TOPP4* (Figure 6D). We also detected the TOPP4 protein level in the *gid1a/b/c* mutant treated with or without GA_3_. Compared to wild type, TOPP4 protein was significantly decreased in the *gid1a/b/c* mutant (Figure 6E), probably attributed to that GA could not be perceived in it. After GA_3_ treatment, the TOPP4 protein level was not obviously changed in the *gid1a/b/c* mutant (Figure 6E). These results indicated that GA enhances the TOPP4 protein level through a GA-GID1 pathway.

### TOPP4 Acts Upstream of RGA and GAI in GA Signal Transduction

**Figure 7:**
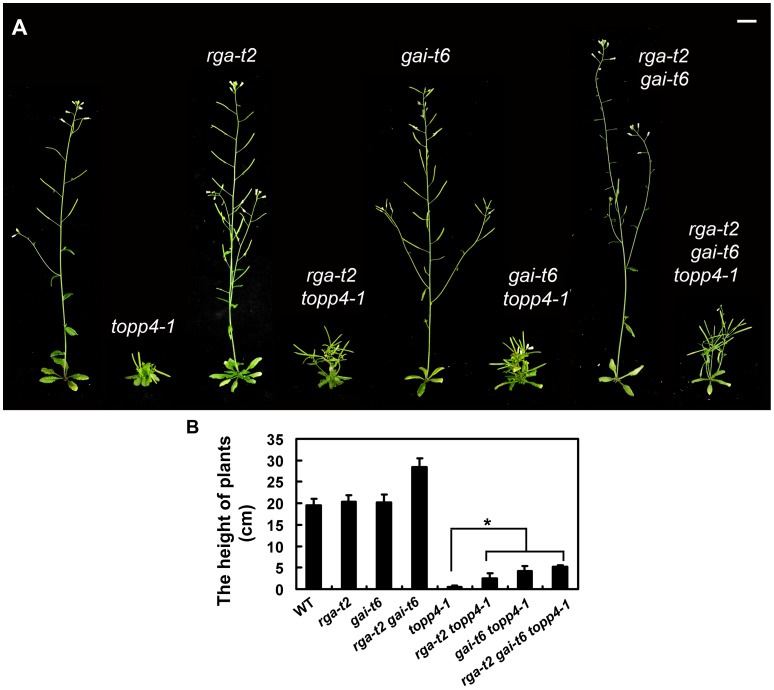
Loss-of-function mutants *rga-t2* and *gai-t6* can partially suppress the dwarfed phenotype of *topp4-1*. (**A**) Representative 5-week-old wild-type, *topp4-1*, *rga-t2*, *rga-t2 topp4-1*, *gai-t6*, *gai-t6 topp4-1*, *rga-t2 gai-t6*, and *rga-t2 gai-t6 topp4-1* plants. The *rga-t2 topp4-1*, *gai-t6 topp4-1*, and *rga-t2 gai-t6 topp4-1* double and triple mutants were back-crossed with Col six times. The *rga-t2*, *gai-t6* single mutants and the *rga-t2 gai-t6* double mutant screened from the same genetic cross were used as control. Scale bars = 1 cm. (**B**) The height of 6-week-old wild-type, *rga-t2*, *gai-t6*, *rga-t2 gai-t6*, *topp4-1*, *rga-t2 topp4-1*, *gai-t6 topp4-1*, and *rga-t2 gai-t6 topp4-1* plants. Asterisk represents statistic differences based on Student's *t* test with P<0.05. Error bars represent SE (n = 20).

RGA and GAI are the main repressors of GA signaling and their accumulation causes severe dwarf phenotypes in plants [44]. Given the fact that *topp4-1* also showed severe dwarfism, genetic interactions between TOPP4 and RGA or GAI were studied. We screened *rga-t2 topp4-1*, *gai-t6 topp4-1*, and *rga-t2 gai-t6 topp4-1* from offsprings of a cross between *topp4-1* and the DELLA penta mutant (*gai-t6 rga-t2 rgl1-1 rgl3-1 SGT625-5-2*, L*er* background). Then, the *rga-t2 topp4-1*, *gai-t6 topp4-1*, and *rga-t2 gai-t6 topp4-1* double and triple mutants were back-crossed with Col six times for subsequent analyses. At the same time, the *rga-t2* and *gai-t6* single mutants and the *rga-t2 gai-t6* double mutant were screened from the same genetic cross as control (Figure 7A). Phenotypic analyses revealed that the loss-of-function mutants *rga-t2* and *gai-t6* could partially reverse the defective phenotype of *topp4-1* (Figure 7A). The *topp4-1* mutant had almost no inflorescences, but both *rga-t2 topp4-1* and *gai-t6 topp4-1* double mutants had 2–3 cm inflorescences, relatively longer than those of *topp4-1* (Figure 7A–B). And the triple mutant *rga-t2 gai-t6 topp4-1* was taller than the double mutants (Figure 7A–B). These results suggested that RGA and GAI are repressors of TOPP4-mediated stem elongation. TOPP4 therefore may genetically associate with RGA and GAI in the GA signaling pathway.

We also analyzed the relationship between TOPP4 and three other DELLA proteins, RGL1, RGL2, and RGL3. The *rgl* mutations failed to rescue the dwarfism of *topp4-1* (Figure S8A). Previous studies provided evidence that RGL1, RGL2, and RGL3 may not be required for the repression of stem elongation, but may be mainly involved in seed germination and floral development [9], [12]–[14]. We next examined the flower development and seed germination in those double mutants. The defective flower morphology of *topp4-1* was also observed in *rgl1-1 topp4-1*, *rgl2-1 topp4-1*, and *rgl3-1 topp4-1* (Figure S8B–C). The seed germination of *rgl1-1 topp4-1* and *rgl3-1 topp4-1* was slightly resistant to paclobutrazol (PAC, a specific inhibitor blocking the kaurene oxidase reaction in GA biosynthetic pathway), similar to that of *topp4-1*, whereas *rgl2-1 topp4-1* had more resistance to PAC, similar to the single mutant *rgl2-1* (Figure S8D) [9]. Therefore, it seemed that TOPP4 has no genetic interaction with RGL1, RGL2, and RGL3, regarding stem elongation, flower development, or seed germination.

### TOPP4 Promotes GA-Induced Degradation of DELLA Proteins

**Figure 8:**
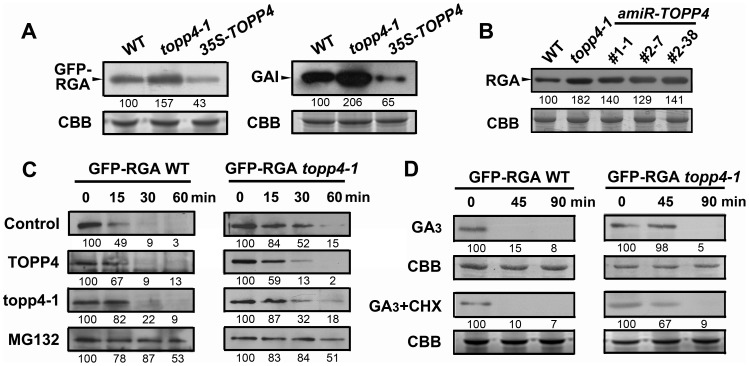
TOPP4 controls the stability of DELLA proteins. (**A**) The protein levels of GFP-RGA and GAI in wild-type, *topp4-1*, and *TOPP4* overexpressing plants determined by immunoblotting using antibody against GFP or GAI. (**B**) The protein levels of RGA in wild-type, *topp4-1*, and three *amiR-TOPP4* plants. (**C**) *In vitro* degradation of GFP-RGA. Total protein extractions from wild-type and *topp4-1* plants were incubated with TOPP4, topp4-1, or 100 µM MG132 for the indicated time. (**D**) The degradation of GFP-RGA in wild-type and *topp4-1* plants induced by 100 µM GA_3_ or 100 µM GA_3_ together with 50 µM CHX. The time refers to the period that seedlings were treated with GA_3_ or GA_3_ and CHX before immunoblotting analysis. Numbers under lanes indicate relative band intensities that were quantified and normalized for each panel. The coomassie brilliant blue-stained RbcS protein was used as loading controls.

From the genetic results, we hypothesized that RGA and GAI could be over-accumulated in *topp4-1*. To test this hypothesis, the levels of GFP-RGA and GAI in wild type, *topp4-1*, and *TOPP4* overexpressing transgenic plants were assessed by immunoblotting. The *topp4-1* plants had more RGA and GAI than wild type, whereas these proteins were significantly lower in *TOPP4* overexpressing plants than in wild type (Figure 8A). At the same time, the RGA protein level was also significantly increased in three amiRNA lines (Figure 8B). Thus, TOPP4 may positively regulate the degradation of RGA and GAI and the dwarfed phenotype of *topp4-1* may be caused by overaccumulation of these two proteins.

The degradation of DELLA proteins required dephosphorylation at Ser or Thr [36], but the phosphatase responsible for this activity had not been identified. We considered that TOPP4 is likely a candidate for this process. This was tested by comparing the degradation of GFP-RGA in wild-type plants and the *topp4-1* mutant. First, a previously reported cell-free system [36] was used in which total proteins were solubilized in a degradation buffer and incubated at 22°C for 0, 15, 30, and 60 min followed by determination of the GFP-RGA abundance by immunoblotting. GFP-RGA protein from wild-type plants was rapidly degraded after 15 min of incubation and little was detected after 60 min (Figure 8C). Degradation rate was clearly slower in the *topp4-1* mutant (Figure 8C). Addition of TOPP4 protein immunoprecipitated from wild-type plants to total protein extracts of the *topp4-1* mutant increased the rate of GFP-RGA degradation, although not to the level of wild type (Figure 8C). Mutated topp4-1 protein from the *topp4-1* mutant did not reverse the delayed degradation (Figure 8C). These results demonstrated that TOPP4 regulates the stability of DELLA protein.

Carbobenzoxy-Leu-Leu-leucinal (MG132), a specific 26S proteasome inhibitor, is reported to block the degradation of DELLA proteins [24]. We used this inhibitor in this cell-free system to determine if the TOPP4-mediated degradation of DELLA protein is dependent on the ubiquitin-proteasome pathway. Supplementation with 100 µM MG132 strongly blocked the degradation of GFP-RGA both in wild type and *topp4-1* (Figure 8C), suggesting that the 26S proteasome acts downstream of TOPP4 in the degradation of DELLA protein.

GA can rapidly induce DELLA protein degradation [19], so we tested this effect in the *topp4-1* background. Seedlings were incubated in Murashige-Skoog (MS) liquid medium supplemented with 100 µM GA_3_ for 0, 45, and 90 min. Total proteins were extracted from these seedlings and assessed by immunoblotting analyses. GFP-RGA accumulation was rapidly reduced after 45 min treatment with GA_3_ in wild-type seedlings (Figure 8D), but this process was apparently delayed in the *topp4-1* seedlings (Figure 8D), confirming that TOPP4 is critical for the GA-induced degradation of DELLA proteins. When seedlings were treated with both GA_3_ and cycloheximide (CHX, a chemical blocking protein synthesis), the degradation of GFP-RGA was still delayed in *topp4-1* (Figure 8D), suggesting that this phenomenon was not affected by de novo protein synthesis. Taken together, we concluded that TOPP4 facilitates the GA-induced degradation of DELLA proteins through a 26S proteasome pathway.

### TOPP4 Physically Interacts with RGA and GAI *In Vitro* and *In Vivo*

**Figure 9:**
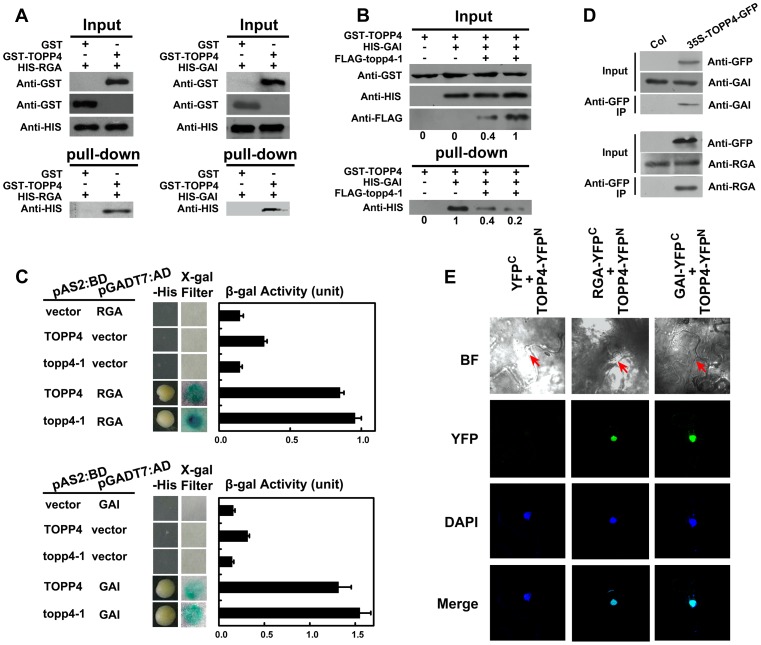
TOPP4 can interact with RGA and GAI both *in vitro* and *in vivo*. (**A**) Pull-down assays were performed to determine the interactions between GST-TOPP4 and HIS-RGA or HIS-GAI. Co-precipitated HIS-RGA or HIS-GAI was analyzed by anti-HIS antibody. (**B**) Pull-down assay was used to test the competitive binding to GAI between TOPP4 and topp4-1 proteins. GST bound GAI protein was analyzed by anti-HIS antibody. Numbers under lanes indicate relative band intensities quantified and normalized for each panel. (**C**) Yeast two-hybrid assays were used to determine the interactions between TOPP4/topp4-1 and RGA or GAI. Quantitative measurements of β-gal activities are shown on the right. Error bars represent SE (n = 3). Mutated topp4-1 seemed to interact with RGA or GAI slightly more than did the wild-type TOPP4. (**D**) Co-immunoprecipitation of RGA or GAI with TOPP4-GFP. Total protein extracts of *35S-TOPP4-GFP* plants were immunoprecipitated with an anti-GFP antibody and detected by immunoblotting using antibody against RGA or GAI. Immunoprecipitation by anti-GFP in Col was used as a negative control. (**E**) BiFC analyses of TOPP4-RGA and TOPP4-GAI interactions in *Nicotiana benthamiana* epidermal cells. BF, bright field; YFP, YFP fluorescence; DAPI, DAPI staining; Merge, merged view of the YFP and DAPI images. Red arrows in BF indicate the location of nucleus. Negative control without YFP fluorescence is shown on the left.

The nuclear localization of TOPP4 suggests a role for regulating nuclear proteins such as transcription factors. DELLA proteins also function in the nucleus and therefore they may physically interact to each other. To test this possibility, we performed a protein-protein interaction assay. Because only RGA and GAI showed genetic relevance with TOPP4, we then only examined interactions of TOPP4 with RGA or GAI proteins both *in vitro* and *in vivo* using a number of different biochemical approaches. Recombinant Histidine (HIS)-RGA, HIS-GAI, and glutathione *S*-transferase (GST)-TOPP4 were purified from *E. coli* and an *in vitro* pull-down experiment was carried out. HIS-RGA or HIS-GAI was pulled down together with GST-TOPP4 using a glutathione sepharose 4B resin (Figure 9A). However, this GST bound GAI protein was gradually reduced when the amount of FLAG-topp4-1 was increased in the same reaction system (Figure 9B), indicating that mutated topp4-1 protein and TOPP4 can competitively interact with DELLA proteins. Next, TOPP4 or topp4-1 was expressed as DNA binding domain (BD) protein fusions, and RGA and GAI were expressed as transactivation domain (AD) protein fusions in yeast strain Y190. Interactions of TOPP4-BD and RGA-AD or GAI-AD were confirmed by β-galactosidase (β-gal) activity (Figure 9C). Mutated topp4-1 seemed to interact with RGA or GAI slightly more than did the wild-type TOPP4 in yeast two-hybrid assay (Figure 9C). Further, to determine the interaction of TOPP4 and RGA or GAI *in planta*, we performed co-immunoprecipitation (co-IP) and bimolecular fluorescence complementation (BiFC) assays. We used *35S-TOPP4-GFP* plants as materials and Col as a negative control in co-IP assay. TOPP4 protein was immunoprecipitated with anti-GFP antibody and TOPP4-bound proteins were subjected to immunoblotting analysis. Both RGA and GAI were co-immunoprecipitated with TOPP4 in *35S-TOPP4-GFP*, but could not be detected in immunoprecipitated complexes of Col (Figure 9D). Finally, when TOPP4-YFP^N^ and RGA-YFP^C^ or GAI-YFP^C^ were transiently co-expressed in leaves of *Nicotiana benthamiana*, YFP fluorescence was clearly detected in nuclei, which were confirmed by 4,6-diamidino-2-phenylindole (DAPI) staining (Figure 9E). These results strongly supported the existence of *in vitro* and *in vivo* interactions between TOPP4 and the two DELLA proteins RGA and GAI.

### Phosphorylated RGA and GAI Are the Substrates of TOPP4

**Figure 10:**
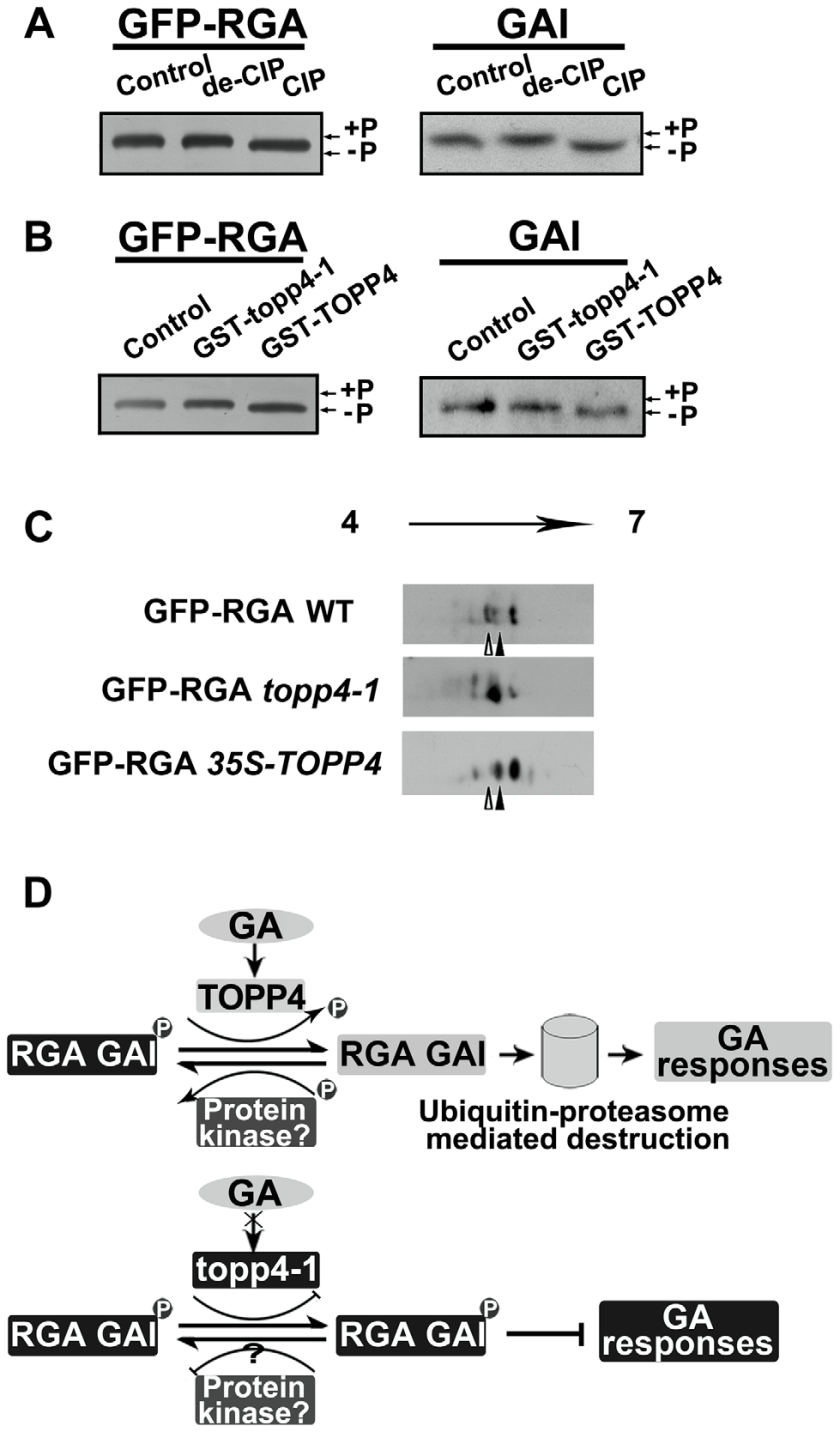
DELLA proteins are direct substrates of TOPP4. (**A**) Immunoblotting assays of GFP-RGA and GAI proteins incubated with denatured CIP (de-CIP) or CIP. (**B**) Immunoblotting assays of GFP-RGA and GAI proteins incubated with GST-topp4-1 or GST-TOPP4 from *E. coli* suggested that TOPP4, but not topp4-1, can directly dephosphorylate phosphorylated RGA and GAI. Phosphorylated status, +P; dephosphorylated status, −P. (**C**) 2-DE analyses of post-translational modification of GFP-RGA in wild-type, *topp4-1*, and *TOPP4* overexpressing plants. The phosphorylation status of RGA was increased in *topp4-1* while the dephosphorylation status of RGA was increased in *35S-TOPP4* plants compared to wild type. The total protein extractions were separated by 2-DE and immunoblotted with anti-GFP antibody. (**D**) A current model of TOPP4 function on DELLA stability in the GA signaling pathway.

We next asked whether phosphorylated RGA and GAI could be dephosphorylated by TOPP4. Immunoblotting analysis using anti-GFP antibody resulted in a single band of RGA (Figure 10A). After treatment with active calf intestinal phosphatase (CIP), the band had greater electrophoretic mobility, which is representative of the dephosphorylated form (Figure 10A). Similar results were obtained for GAI protein (Figure 10A). This finding was consistent with the GAI phosphorylation status reported by Fu et al. [22]. To further demonstrate that phosphorylated RGA and GAI are the substrates of TOPP4, we incubated total protein extractions from the *topp4-1* mutant with GST-TOPP4 or mutated GST-topp4-1 produced by *E. coli*. Both GFP-RGA and GAI treated with GST-TOPP4, but not GST-topp4-1, showed increased electrophoretic mobility (Figures 10B and S9A). These results are consistent with those obtained by CIP treatment, indicating that TOPP4, but not topp4-1, can dephosphorylate phosphorylated RGA and GAI directly.

Moreover, we analyzed the post-translational modification of GFP-RGA in wild type and the *topp4-1* mutant by two-dimensional gel electrophoresis (2-DE). Protein gel blots showed several spots with different isoelectric point (pI) values in wild-type, *topp4-1*, and *35S-TOPP4* transgenic plants. In wild type, the basic forms of GFP-RGA, which represent the dephosphorylated status, were dominant (Figures 10C and S9B), while in *topp4-1*, the basic forms were decreased, and the acidic forms, which represent the phosphorylated status, were increased compared to wild type (Figures 10C and S9B). In *TOPP4* overexpressing plants, the basic forms of GFP-RGA were increased significantly. The spot at the more acidic side (indicated by open arrowhead) was decreased while that at the more basic side (indicated by solid arrowhead) was increased compared to wild type (Figure 10C). These results provided further confirmation that TOPP4 can dephosphorylate DELLA proteins. The phosphorylated forms of RGA were increased in *topp4-1*, along with its slow degradation and high accumulation in the mutant (Figure 8), suggesting that the dephosphorylated forms of DELLA proteins may facilitate their destruction.

In addition, to investigate whether TOPP4 can dephosphorylate DELLA protein in the absence of GA, we transformed *35S-TOPP4* into *gai-1*, a mutant in which GAI cannot be degraded through GA-GID1 for the deletion of its DELLA domain [44]. The result showed that overexpression of *TOPP4* could not rescue the dwarfed phenotype of *gai-1* (Figure S10), suggesting that TOPP4-mediated DELLA dephosphorylation is dependent on the formation of the GA-GID1-DELLA complex.

## Discussion

Reversible phosphorylation and dephosphorylation, controlled by protein kinases and protein phosphatases, respectively, is one of the most important mechanisms of the post-translational modifications of proteins. Previous studies revealed the crucial role of dephosphorylation in plant development, mediated by a protein phosphatase 2A (PP2A), a protein phosphatase 2C (PP2C), and a protein phosphatase 6 (PP6) [48]–[51]. However, the regulatory functions of PP1s in plant development are poorly understood. In this study, we report the isolation and phenotypic characterization of a *topp4-1* mutant identified from EMS-mutagenized *Arabidopsis* plants. Our results suggested a positive role of TOPP4 in the GA signaling pathway, through regulating the stability of DELLA proteins.

Protein kinases and phosphatases play important roles in several phytohormonal signaling pathways in plants. For example, in BR signaling, brassinosteroid-insensitive 2 (BIN2) phosphorylates and inactivates the transcription factor brassinazole-resistant 1 (BZR1) to inhibit plant growth, whereas PP2A dephosphorylates BZR1-P and promotes or inhibits the expression of its downstream response genes [49]. In ABA signaling, PP2C dephosphorylates SNF1-related protein kinase 2s (SnRK2s) to block ABA-mediated stress responses [50]. A key enzyme in ethylene biosynthesis pathway, 1-aminocyclopropane-1-carboxylic acid synthase (ACS), is stabilized by phosphorylation by mitogen-activated protein kinase 6 (MPK6) and destabilized by dephosphorylation by PP2A [52], [53]. In GA signal transduction, however, the function of protein phosphorylation on the stability of DELLA proteins has remained controversial. Fu et al. [54] revealed that both protein kinases and protein phosphatases were required for the GA-induced degradation of the barley DELLA protein SLENDER (SLN1). Subsequent studies indicated that protein phosphorylation increased the interaction between DELLA proteins and SCF ubiquitin ligase [21], [22], [55]. Conversely, Itoh suggested that phosphorylation of SLENDER RICE1 (SLR1) was independent of its degradation in rice [56]. However, recent work showed that Ser/Thr phosphatase inhibitors suppressed the degradation of RGL2 and RGA in *Arabidopsis* [36], [57]. More recently, a rice casein kinase I named as early flowering 1 (EL1), was identified and shown to stabilize the rice DELLA protein SLR1 by phosphorylation [37].

Based on our genetic and biochemical data and previous studies [36], [57], we concluded that the stability of RGA and GAI is regulated by protein phosphorylation and dephosphorylation (Figure 10D). The phosphorylated forms of RGA and GAI are stable and active, inhibiting the GA signaling pathway in *Arabidopsis*, consistent with the action of SLR1 in rice [37]. TOPP4 dephosphorylates the phosphorylated RGA and GAI, targeting them for the GA-induced degradation by the ubiquitin-proteasome pathway to promote stem elongation. Degradation of RGA and GAI relieves their restraint on GA signaling. In this process, GA promotes TOPP4 protein accumulation through GID1 (Figure 6C,E), thereby enhancing the dephosphorylation and degradation of DELLAs. But in the *topp4-1* mutant, GA cannot promote the accumulation of mutated topp4-1 protein and topp4-1 cannot dephosphorylate RGA and GAI (Figures 6C and 10B). The phosphorylated RGA and GAI are degraded slowly in response to GA_3_ (Figure 8D), and their accumulation blocks GA signal transduction, resulting in GA-related mutant phenotypes, especially severe dwarfism. In *TOPP4* overexpressing plants, less RGA and GAI accumulate than in wild-type plants due to excessive dephosphorylation, leading to a taller inflorescence (Figures 2F and S6). Therefore, our data indicated that TOPP4 is a positive regulator of the GA signaling pathway and functions in stimulating stem elongation by destabilizing DELLA proteins. This dephosphorylation process is likely dependent on the formation of GA-GID1-DELLA module, because overexpression of *TOPP4* could not rescue the dwarfed phenotype of *gai-1* (Figure S10).

However, unlike some other phosphorylated proteins [BR-signaling kinase 1 (BSK1)] with many phosphorylation sites in plants [58], RGA protein may have very few phosphorylation sites, which was supported by the results of 2-DE (Figures 10C and S9B). Our dephosphorylation analyses showed that the difference between phosphorylated and dephosphorylated status of DELLA proteins was very little (Figure 10A,B), and their status is therefore difficult to assess. This might be one of the reasons that the protein phosphorylation-dephosphorylation modification of DELLAs has been controversial.

An important finding in this work is that the *topp4-1* mutant, with a 246Thr to 246Met amino acid substitution attributed to a G-to-A single nucleotide alteration, displays severe growth defects. The 246Thr is not a conservative site in all TOPPs (Figure S11). Its mutation does not affect the interaction of topp4-1 with DELLA proteins (Figure 9C), and the mutated topp4-1 and TOPP4 can competitively interact with DELLA proteins (Figure 9B). But the mutation impairs the dephosphorylation function of topp4-1 on DELLAs (Figure 10B). This result can explain that the single mutation of *TOPP4* causes a dominant-negative effect on plant growth and development, resulting in severe defects in *topp4-1*. The dominant-negative effect is further confirmed by genetic data: expressing either 35S-*topp4-1* or *pTOPP4-topp4-1* in wild type could mimic the *topp4-1* mutant phenotypes (Figure 2A). *pTOPP4-TOPP4* only very slightly reversed the defects of *topp4-1* and even *35S-TOPP4* could not completely recover it (Figures S3 and 3A). And knocking down *topp4-1* gene in the *topp4-1* mutant could partially rescue the deficient phenotypes (Figure 2C). Therefore, this dominant-negative material is crucial for elucidating the distinct functions of TOPPs in *Arabidopsis*.

There are nine PP1s (TOPP1–TOPP9) in *Arabidopsis* [40], [59], and they share 90.9–99.7% amino acid similarities (Figure S11) [60]. It seems that there might be a high degree of functional redundancy among them. In this study, we demonstrated that the amiRNA lines of *TOPP4* showed dwarfed phenotypes, with overaccumulated RGA protein (Figures 3C,3D,8B). In those amiRNA lines, both *TOPP4* gene expression and TOPP4 protein level were dramatically reduced (Figure 3E–F). These results confirmed that TOPP4 is a major protein phosphatase in regulating GA-mediated DELLA protein degradation in *Arabidopsis*.

The PP1 catalytic subunit often binds the regulatory subunit to form a functional enzyme. These regulatory subunits determine the catalytic activity, target the catalytic subunit to specific subcellular compartment, and modulate the specificity of substrates [61]. There are about 100 predicted PP1-binding regulatory subunits in animals [62]. However, to date, only inhibitor-3 (inh3), *Arabidopsis* I-2 (AtI-2), PP1 regulatory subunit2-like protein 1 (PRSL1) have been identified in plants [41], [42], [63]. In this study, we found that TOPP4 can directly bind RGA and GAI proteins. However, these two proteins have neither PVxF motif nor SILK motif which are present in PP1 regulatory subunits [64]. It is likely that TOPP4 may require another unknown regulatory subunit for controlling the dephosphorylation of RGA and GAI *in vivo*. *TOPP4* is ubiquitously expressed in various organs throughout different growth stages. Therefore, it may be involved in regulation of many developmental processes. The DELLA deficient mutants *rga-t2* and *gai-t6* only partially rescued *topp4-1* phenotypes, suggesting that TOPP4 may promote plant growth also through other signaling pathways. The plasma membrane-localization of TOPP4 also implies that it may participate in many other signal transductions. Identification of the regulatory subunits of TOPP4 in different subcellular locations, tissues, and development stages of plants may provide significant insights into the molecular mechanism of this protein on plant development.

To conclude, we have identified a key phosphatase that can directly dephosphorylate DELLA proteins in *Arabidopsis*; and we elucidated a mechanism of TOPP4 in regulating GA-mediated stem elongation by controlling DELLA protein stability. Future work will focus on identification of a protein kinase involved in phosphorylating *Arabidopsis* DELLA proteins, the specific phosphorylation sites on DELLA proteins regulating their stability, and the roles of TOPP4 in other developmental processes.

## Materials and Methods

### Plant Materials

EMS-mutagenized *Arabidopsis thaliana* (L.) Heynh transgenic line *E361-1* was screened for mutant *topp4-1*. After back-crossing three times with the wild-type Col-0, *topp4-1* plants were used for subsequent research. The T-DNA insertion mutant lines, N466328 and SALK_090980, were obtained from European *Arabidopsis* Stock Centre (NASC) and *Arabidopsis* Biological Resource Center (ABRC), respectively. Primers used for identifying homozygous lines are indicated in Table S2 on line. The single, double and triple mutants *rga-t2*, *gai-t6*, *rga-t2 gai-t6*, *rga-t2 topp4-1*, *gai-t6 topp4-1*, *rga-t2 gai-t6 topp4-1*, *rgl1-1 topp4-1*, *rgl2-1 topp4-1*, and *rgl3-1 topp4-1* were generated from the cross of *topp4-1* with DELLA penta mutant (*gai-t6 rga-t2 rgl1-1 rgl3-1 SGT625-5-2*, cs16298, from ABRC). Primers used for genotyping are indicated in Table S2 on line. *pRGA-GFP-RGA* line (cs6942), *ga1-3* (cs3104), *ga4* (cs62), *gai-1* (cs63), and *gid1a-2/gid1b-3/gid1c-1* (*gid1a/b/c*, cs16297) were ordered from ABRC. *pRGA-GFP-RGA topp4-1* was generated from the cross of *pRGA-GFP-RGA* with *topp4-1*.

### Map-Based Cloning

The *topp4-1* plants from the F_2_ population of a cross between the *topp4-1* mutant in Col-0 ecotype background and L*er*-0 (cs20, from ABRC) were selected for mapping. Simple sequence length polymorphism (SSLP) markers were used to define the mutant gene to chromosome 2 [65]–[67]. The markers used in fine mapping are listed in the Table S1 on line, including In/Del and CAPS. All of the eight new markers were developed by our lab. We sequenced the 90-Kb between markers T5I7-29008 and T28M21-47168 to identify the *TOPP4* gene finally.

### Plasmid Constructs and Plant Transformation

For the complementation experiment and overexpressing transgenic line *35S-TOPP4*, a 1696-bp genomic sequence consisting of the entire coding region was PCR-amplified by PCR from the genome of wild-type Col-0 with primer set 5′-GGGGTACCTCTTTGCGCGTAATTTTCT-3′ and 5′-CGAGCTCCTCAAGAAAGACCAAATCCA-3′. Underlined regions introduce *Kpn* I and *Sac* I sites, respectively. The amplified fragment was cloned into pCAMBIA 1300. Transgenic lines expressing GFP-tagged TOPP4 (*35S-TOPP4-GFP*), *35S-topp4-1*, and *35S-TOPP4-RFP* were generated by amplifying the cDNA of Col-0 or *topp4-1* with a primer set 5′-TCTAGAATGGCGACGACGACGAC-3′ and 5′-GGTACCTCCTCCTCCAATCTTTGTGGACATCATGA -3′. Underlined regions introduce *Xba* I and *Kpn* I sites, respectively. The amplified fragment was cloned into pCAMBIA 1300-GFP or pCAMBIA 1300-RFP. For *pTOPP4-TOPP4*, the *TOPP4* promoter about 2-Kb upstream of ATG was generated with a primer set 5′-CAAGCTTTTCCGACTTAATCCGGTCCA-3′ and 5′-CTCTAGACCTAATTTTTTCGACCCC-3′. Underlined regions introduce *Hind* III and *Xba* I sites, respectively. The 35S promoter of *35S-TOPP4* was replaced by this amplified fragment. For *pTOPP4-topp4-1*, the 35S promoter of *35S-topp4-1* was replaced by the *TOPP4* promoter. For promoter analysis (*pTOPP4-GUS*), the promoter was generated with a primer set 5′-CAAGCTTTTCCGACTTAATCCGGTCCA-3′ and 5′-CGGGATCCCCTAATTTTTTCGACCCC-3′. Underlined regions introduce *Hind* III and *Bam*H I sites, respectively. The amplified fragment was cloned into pCAMBIA 1300-GUS that we reconstructed. For transient expression in *Arabidopsis* protoplasts, *TOPP4-YFP* were generated by amplifying the cDNA of *TOPP4* with a primer set 5′-GTCGACATGGCGACGACGACGAC -3′ and 5′-GGATCCAATCTTTGTGGACATCATGA -3′. Underlined regions introduce *Sal* I and *Bam*H I sites, respectively. The amplified fragment was cloned into PA7-YFP.

Primers for *amiRNA-TOPP4* were designed by Web MicroRNA Designer 3 (http://wmd3.weigelworld.org/cgi-bin/webapp.cgi) oligo design algorithm using the RS300 vector sequence and the amiRNA sequences of *TOPP4* gene (*amiR-TOPP4*-1: 5′-TACCTAATTTTTTCGACGCCA-3′; *amiR-TOPP4*-2: 5′-TAAAATTACGCGCAAAGACTA-3′). Detailed information using overlapping PCR and primer sets is available at Web MicroRNA Designer 3 web site. The full amiRNA fold-back fragment was subsequently cloned into pCAMBIA 1300 by X*ba* I/K*pn* I. The alignment of the nucleotide sequence for targets of *amiR-TOPP4* with the same regions of other *TOPPs* in *Arabidopsis* was presented in Figure S12.

Plant transformation plasmids were introduced into *Agrobacterium tumefaciens* strain GV3101, and then were transformed into *Arabidopsis* plants using the flower-dipping method [46]. T_1_ transgenic lines were selected on MS [68] plates with 25 mg/L hygromycin (Solarbio, Beijing, China). Genetic and phenotypic analyses were performed mainly in the T_2_ generation.

### GUS Staining and GFP Localization

The T_2_ transgenic plants carrying the *pTOPP4-GUS* construct were immersed in GUS staining solution [50 mM Na-Phosphate buffer, pH 7.0, 1 mM EDTA, 0.1% Triton X-100, 100 µg/mL chloramphenicol, 1 mg/mL 5-bromo-4-chloro-3-indolyl-β-D-glucuronic acid (X-gluc), 2 mM ferricyanide, 2 mM ferrocyanide] and incubated overnight at 37°C. Then, they were decolorized in chloral hydrate solution [8 g of chloral hydrate, 1 mL of glycerol, and 2 mL of water]. The stained tissues were observed and photographed by light microscopy (80*i*, Nikon). Subcellular localization of TOPP4-GFP was photographed by confocal microscopy (Olympus FluoView FV1000MPE). For plasmolysis studies, roots of 10-day-old *35S-TOPP4-GFP* transgenic plants were analyzed as described previously [69] and observed by confocal microscopy.

### qRT-PCR

All qRT-PCR measurements were performed using a MX 3000 Real-time PCR system (Stratagene, La Jolla, CA) with SYBR Premix Ex Taq (Takara Bio, Inc., Shiga, Japan). Total RNA (E.Z.N.A Plant RNA Kit, OMEGA Bio, tek, Norcross, GA) was extracted from 0.05 g of tissue from 2-week-old seedlings grown on MS medium or MS medium containing 10 µM GA_3_ (Sigma, St. Louis, MO). The cDNAs were synthesized from 1 µg of total RNA using the PrimeScript RT reagent Kit (Perfect Real Time) (Takara Bio, Inc., Shiga, Japan). We used the housekeeping gene *GLYCERALDEHYDE-3-PHOSPHATE DEHYDROGENASE C SUBUNIT* (*GAPC*) as a normalization control. All experiments were performed with three replicates. The primers used for qRT-PCR are listed in Table S2 on line.

### Transient Expression, GA_3_ Treatment, and Seed Germination

For transient expression in *Arabidopsis* protoplasts, mesophyll protoplast separation and PEG4000-mediated transfection were performed as previously described [70]. *TOPP4-YFP* was used in this process. YFP signals were observed with a confocal microscopy. For transient expression in *Nicotiana benthamiana* leaves, the *Agrobacterium* strain containing *35S-TOPP4-GFP* construct was infiltrated into leaves of 4-week-old tobacco plants, and GFP and RFP were observed 2 days after transformation by a confocal microscopy.

For inflorescence analyses, 7-day-old plants grown in soil were sprayed with 100 µM GA_3_ or water for control every 3 days for 3 weeks. For seed germination, seeds were grown on the MS plates contained 0, 5, and 10 µM PAC. Seed germination was scored 6 days after vernalization. Thirty to fifty seedlings were measured each time. These experiments were repeated three times independently.

### Antibodies and Immunoblotting Assay

The primary antibodies used in this study were anti-RGA (Agrisera, Vännäs, Sweden), anti-GFP (Invitrogen, Carlsbad, CA), anti-GST (ZSGB-Bio, Beijing, China), anti-HIS (ZSGB-Bio, Beijing, China), anti-TOPP4, and anti-GAI. Anti-TOPP4 and anti-GAI were made in our lab. Anti-GAI was generated in rabbits by immunizations with full-length protein sequence. The anti-TOPP4 antibodies were generated in rabbits using 150 amino acids at the N terminal of TOPP4. Both anti-GAI and anti-TOPP4 antibodies were affinity purified, and the specificity of them were determined using mutants *gai-t6* and N466328, respectively. Immunoblotting analysis was performed as previously described [71]. For immunoblotting detection of TOPP4 in plasma membrane, plasma membrane extraction was isolated from 2-week-old wild-type seedlings and anti-GFP and anti-PIN1 antibodies (N782245, NASC) were used for detecting TOPP4 and PIN1 protein, respectively. For DELLA protein level assay, 20-day-old wild-type, *topp4-1*, *35S-TOPP4*, and three amiRNA transgenic plants were used. For DELLA protein degradation assay, total protein extracts from 20-day-old *pRGA-GFP-RGA* and *pRGA-GFP-RGA topp4-1* plants were prepared as previously described [36] and treated with TOPP4 immunoprecipitated from wild-type plants using anti-TOPP4 antibody, topp4-1 from the *topp4-1* mutant, or 100 µM MG132 (Calbiochem, Darmstadt, Germany). For GA_3_ treatment, 20-day-old *pRGA-GFP-RGA* and *pRGA-GFP-RGA topp4-1* plants were incubated in MS liquid medium containing 100 µM GA_3_ or 100 µM GA_3_ together with 50 µM CHX for the indicated time periods. For TOPP4 protein level analysis, 2-week-old wild-type, *topp4-1*, *gid1a/b/c* or *35S-TOPP4-GFP* plants were treated with 10 or 100 µM GA_3_ for 4 h. The coomassie brilliant blue-stained rubisco small subunit (RbcS) protein was used as a loading control as indicated. Each experiment was repeated at least three times. Relative band intensities were then calculated for each immunoblot panel by Emage J.

### Yeast Two-Hybrid Assay

The yeast strain Y190 was used in our experiments. Yeast transformations were performed according to the MATCHMAKER two-hybrid system 3 (Clontech, Shiga, Japan). Full-length cDNA of *TOPP4* or *topp4-1* fused to the DNA-binding domain of GAL4 was used as the bait protein and *GAI* or *RGA* fused to the transcriptional activation domain of GAL4 was used as the prey protein. Yeast clones containing the *GAL4-BD-TOPP4/GAL4-BD-topp4-1* and *GAL4-AD-GAI* or *GAL4-AD-RGA* constructs were plated on synthetic dextrose (SD)-His-Trp-Leu medium for 5 days at 30°C to assay for interaction. The results of β-gal filter assay were observed in one hour. β-gal activity were detected according to the manufactures protocol (Clontech, Shiga, Japan). This experiment was repeated at least three times.

### *In Vitro* Pull-Down Assay and *In Vivo* Co-IP Assay

The GST-TOPP4, HIS-GAI, HIS-RGA proteins were expressed in *E. coli* BL21. The recombinant proteins were co-incubated in the presence of glutathione sepharose 4B resin (GE, Fairfield, CT), which was used to selectively bind the GST fusion proteins with PBS buffer [140 mM NaCl, 2.7 mM KCl, 10 mM Na_2_HPO_4_, 1.8 mM KH_2_PO_4_]. The bound proteins were eluted with 1× sodium dodecyl sulfate (SDS) loading buffer and analyzed with anti-GST and anti-HIS antibodies. For competitive pull-down assay, FLAG-topp4-1 was expressed in *E. coli* BL21. Pull-down reactions were performed in the presence of 5 µg GST-TOPP4, 2.5 µg HIS-GAI, and 1.25 µg or 2.5 µg FLAG-topp4-1. HIS-GAI and FLAG-topp4-1 were mixed, pulled-down with GST-TOPP4 and detected by anti-HIS antibody [72]. These experiments were repeated at least three times.

Co-immunoprecipitation studies of TOPP4 and RGA or GAI were performed on 10-day-old seedlings of Col and *35S-TOPP4-GFP*. RGA and GAI proteins from these two materials were adjusted to the same amount. Immunoprecipitation of TOPP4 protein used anti-GFP antibody. Protein G agarose (GE, Fairfield, CT) was used to precipitate the immunoprotein complexes with IP buffer [150 mM NaCl, 50 mM Tris-HCl, pH 7.5, 1% NP-40, 1% protease inhibitors]. After immunoprecipitation, beads were washed four times with IP buffer. Proteins were then released and collected by boiling in 2× SDS loading buffer for 5 min. Immunoprecipitation products were detected by immunoblotting with RGA- or GAI-antibody, respectively. This experiment was repeated at least three times.

### BiFC

The full-length open reading frame sequences for the *Arabidopsis TOPP4*, *RGA*, and *GAI* were amplified and cloned into pEearleygate201-YN and pEearleygate202-YC BiFC vectors to generate TOPP4-YFP^N^, RGA-YFP^C^, and GAI-YFP^C^ [73], [74]. Co-infiltration of *Agrobacterium* strains containing the BiFC constructs and the p19 silencing plasmid was carried out at OD 600 of 0.7∶0.7∶1.0 and infiltrated into leaves of 4-week-old *Nicotiana benthamiana* plants [24]. The BiFC signal was observed 3 days after infiltration using a fluorescence microscope. Leaves were incubated with 0.2 mg/L DAPI for nuclei staining.

### *In Vitro* Dephosphorylation Assay

For CIP treatment, 70 µg of total protein extracts from 20-day-old *topp4-1* seedlings (for GAI assay) and *pRGA-GFP-RGA topp4-1* seedlings (for RGA assay) were added with 50 U CIP (NEB, Ipswich, MA) or the same amount of denatured CIP and incubated at 37°C for 3 h and then detected by immunoblotting. For TOPP4 treatment, 70 µg of total protein extracts from 20-day-old *topp4-1* seedlings (for GAI assay) and *pRGA-GFP-RGA topp4-1* seedlings (for RGA assay) with buffer [50 mM Tris-HCl (pH 7.0), 0.1 mM Na_2_EDTA, 5 mM DTT, 0.01% (w/v) Brij 35, 1 mM MnCl_2_, 1 µM protease inhibitors] were added with 5 µg GST-TOPP4 or GST-topp4-1 and incubated at 30°C for 1 h, after which they were subjected to immunoblotting. An equal amount of extracts was used as the control for each treatment. The reaction was terminated by adding loading buffer. Each experiment was repeated at least seven times.

### 2-DE

Total proteins were extracted from 20-day-old *pRGA-GFP-RGA*, *pRGA-GFP-RGA topp4-1*, and *pRGA-GFP-RGA 35S-TOPP4* plants and separated by 2-DE using a 13-cm, pH 4–7 immobilized pH gradient gel (IPG) strip in Ettan IPGphor 3 Isoelectric Focusing System (GE, Fairfield, CT), and the second-dimensional separation was performed on 8% SDS PAGE gel. The same amount of RGA protein was loaded for each sample. The proteins were detected using anti-GFP antibody. This experiment was repeated three times.

### Accession Numbers

Sequence data from this article can be found in the *Arabidopsis* Genome Initiative or GenBank/EMBL databases under the following accession numbers: *TOPP4* (*At2g39840*), *RGA* (*At2g01570*), *GAI* (*At1g14920*), *RGL1* (*At1g66350*), *RGL2* (*At3g03450*), *RGL3* (*At5g17490*), *TOPP1* (*At2g29400*), *TOPP2* (*At5g59160*), *TOPP3* (*At1g64040*), *TOPP5* (*At3g46820*), *TOPP6* (*At5g43380*), *TOPP7* (*At4g11240*), *TOPP8* (*At5g27840*), *TOPP9* (*At3g05580*), *TOPP1* (*Vicia faba*, *AB038648*), *PP1* (*Oryza sativa*, *OSU31773*), s*er/thr PP1* (*Zea mays*, *ZEAMMB73_175230*), *PPP1CC* (*Homo sapiens*, *HGNC:9283*), *PPP1CC* (*Mus musculus*, *MGI:104872*), *PP2A* (*At1g69960*).

## Supporting Information

Figure S1 Comparison of the phenotypes of the *topp4-1* mutant and GA pathway mutants. (A) Representative 4-week-old L*er*, *ga1-3*, *ga4*, *gai-1*, *gid1a/b/c*, Col, and *topp4-1* plants. Scale bars = 1 cm. (B)–(D) Maximum rosette radius (B), days to flower (C), and chlorophyll content (D) of L*er*, *ga1-3*, *ga4*, *gai-1*, *gid1a/b/c*, Col, and *topp4-1* plants. Asterisks represent statistic differences based on Student's *t* test with P<0.05. Error bars represent SE (n = 20).
(TIF)

Figure S2 The expression levels of *TOPP4* and the protein levels of TOPP4 in wild-type plant and the *topp4-1* mutant. (A) Analysis of the *TOPP4* expression in wild-type and *topp4-1* seedlings by qRT-PCR. The expression level of wild type was set to 1.0. Error bar represents SE (n = 3). (B) The protein levels of TOPP4 in wild-type and *topp4-1* plants determined by immunoblotting using antibody against TOPP4.
(TIF)

Figure S3 Transformed *pTOPP4-TOPP4* into *topp4-1* partially rescued the size of rosette leaves but did not rescue the dwarfed phenotype. Four-week-old *topp4-1* and *pTOPP4-TOPP4 topp4-1* transgenic plants are shown. Scale bar = 1 cm.
(TIF)

Figure S4 Two T-DNA insertion alleles of *TOPP4* were identified from GABI-Kat and SALK T-DNA insertion databases. (A) Five-week-old wild-type, *topp4-1*, N466328, and SALK_090980 plants. (B) and (C) PCR-based analysis was used to identify the homozygous plants of N466328 (B) and SALK_090980 (C). (D) Analysis of the *TOPP4* expression in wild-type, N466328, and SALK_090980 plants by qRT-PCR. The expression level of wild type was set to 1.0. Asterisk represents statistic differences based on Student's *t* test with P<0.05. Error bars represent SE (n = 3). Scale bar = 1 cm.
(TIF)

Figure S5 Phenotype of 2-week-old three *amiR-TOPP4* transgenic lines. Scale bar = 1 cm.
(TIF)

Figure S6 Overexpression of *TOPP4* in wild-type plants increased inflorescence height and enhanced organ size. (A) Representative 7-week-old *35S-TOPP4* transgenic lines. (B) Final height of 7-week-old *35S-TOPP4* transgenic lines. Error bars represent SE (n = 20). (C) Ten-day-old wild-type, *topp4-1*, and *35S-TOPP4* seedlings (from left to right). (D) The hypocotyl length of 10-day-old wild-type, *topp4-1*, and *35S-TOPP4* plants. Error bars represent SE (n = 30). (E) Cross section of inflorescences of wild-type and *35S-TOPP4* plants. (F) Rosette leaves from wild-type and *35S-TOPP4* plants. (G) The area of the second pair of rosette leaves of wild-type and *35S-TOPP4* plants. Error bars represent SE (n = 30). (H) The top of inflorescences of wild-type and *35S-TOPP4* plants. Asterisks in (B), (D) and (G) represent statistic differences based on Student's *t* test with P<0.05. Scale bars = 2 cm in (A); 1 mm in (C) and (E); 1 cm in (F); 3 mm in (H).
(TIF)

Figure S7 GA enhanced the TOPP4 protein level. The protein levels of TOPP4-GFP in *35S-TOPP4-GFP* seedlings treated with or without 100 µM GA_3_.
(TIF)

Figure S8 TOPP4 has no genetic interaction with RGL1, RGL2, and RGL3. (A) Representative 6-week-old *topp4-1*, *rgl1-1 topp4-1*, *rgl2-1 topp4-1*, and *rgl3-1 topp4-1* plants. Scale bar = 1 cm. (B) and (C) Floral buds (B) and flowers (C) of *topp4-1*, *rgl1-1 topp4-1*, *rgl2-1 topp4-1*, and *rgl3-1 topp4-1* plants. Scale bars = 0.5 cm. (D) Seed germination of *topp4-1*, wild-type, *rgl1-1 topp4-1*, *rgl2-1 topp4-1*, and *rgl3 topp4-1* treated with different concentrations of PAC. Error bars represent SE (n = 30).
(TIF)

Figure S9 Dephosphorylation assays of RGA and GAI. (A) Immunoblotting assay of GFP-RGA and GAI proteins incubated with GST-topp4-1 or GST-TOPP4 from *E. coli*. Phosphorylated status, +P; dephosphorylated status, −P. (B) 2-DE analysis of posttranslational modification of GFP-RGA in wild-type and *topp4-1* plants.
(TIF)

Figure S10 Overexpression of *TOPP4* could not rescue the dwarfed phenotype of *gai-1*. (A) Representative 6-week-old *gai-1* and *35S-TOPP4 gai-1* #1 plants. Scale bar = 1 cm. (B) Analysis of the *TOPP4* expression in three representative 35S-*TOPP4 gai-1* T_2_ transgenic lines by qRT-PCR. The expression level of *gai-1* was set to 1.0. Asterisks represent statistic differences based on Student's *t* test with P<0.05. Error bars represent SE (n = 3).
(TIF)

Figure S11 Amino acid sequence comparison among TOPP4, other TOPPs, and the PP1s of *Oryza sative*, *Vicia faba*, *Zea mays*, *Homo sapiens*, and *Mus musculus* showing high similarity to TOPP4. The numbers at the top of the sequences indicate the position of amino acid residues in corresponding proteins. Amino acids shared by all members are shaded in the same color and marked by asterisks. Thr246 in TOPP4 is indicated by arrow.
(TIF)

Figure S12 The alignment of the nucleotide sequence for targets of *amiR-TOPP4* with the same regions of other *TOPPs* in *Arabidopsis*. (A) *amiR-TOPP4*-1. (B) *amiR-TOPP4*-2.
(TIF)

Table S1 New developed In/Del and CAPS markers used for fine mapping in the *topp4-1* mutant.
(DOC)

Table S2 Primers used for genotype identification and qRT-PCR.
(DOC)
