# Supplementary figures and images for: Arabidopsis DELLA Protein Degradation Is Controlled by a Type-One Protein Phosphatase, TOPP4
Source: PLoS Genet. 2014 Jul 10;10(7):e1004464. doi: 10.1371/journal.pgen.1004464 (PMC4091783; doi:10.1371/journal.pgen.1004464)

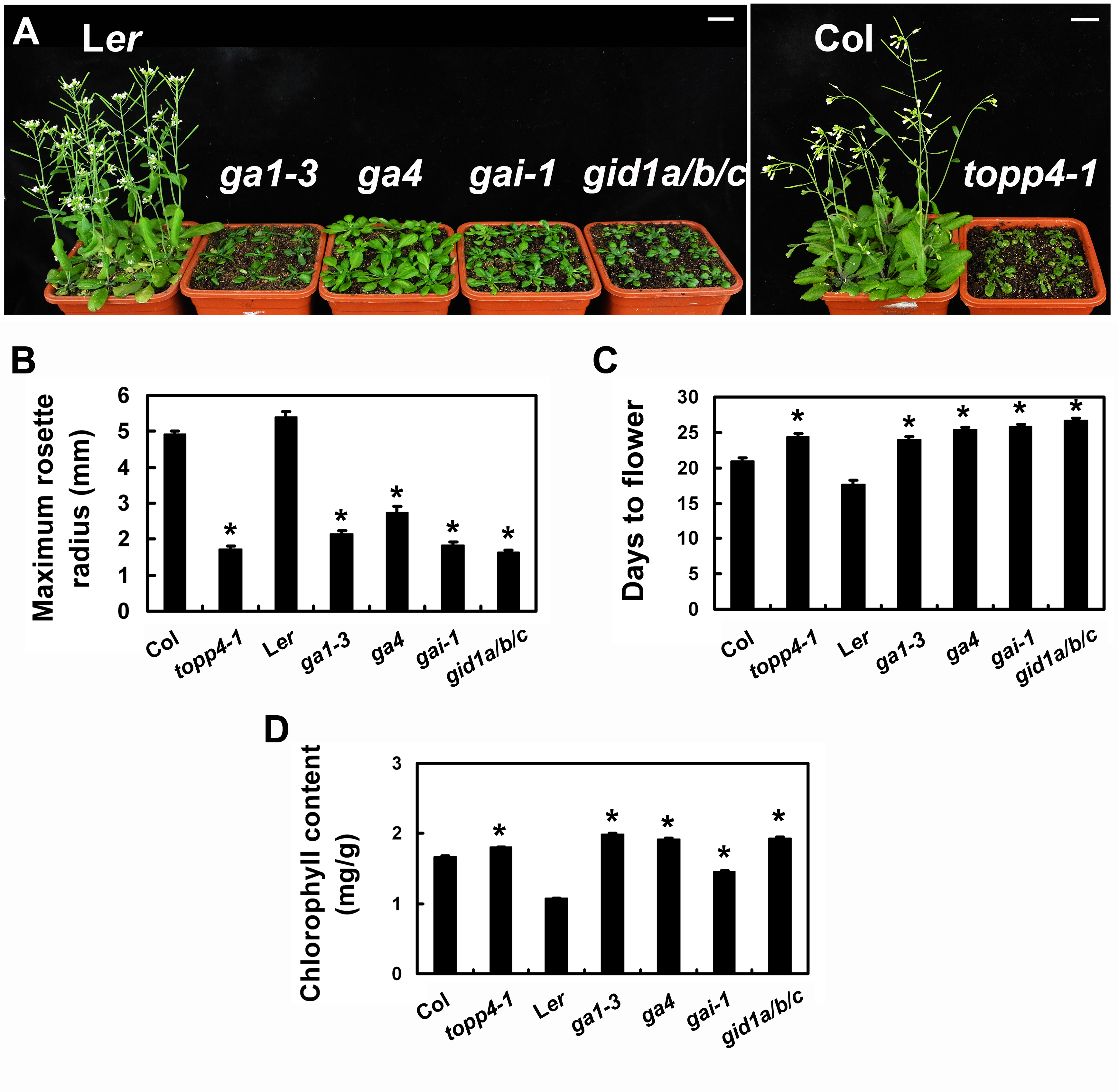

Supplement: Figure S1 — Comparison of the phenotypes of the topp4-1 mutant and GA pathway mutants. (A) Representative 4-week-old Ler, ga1-3, ga4, gai-1, gid1a/b/c, Col, and topp4-1 plants. Scale bars = 1 cm. (B)–(D) Maximum rosette radius (B), days to flower (C), and chlorophyll content (D) of Ler, ga1-3, ga4, gai-1, gid1a/b/c, Col, and topp4-1 plants. Asterisks represent statistic differences based on Student's t test with P<0.05. Error bars represent SE (n = 20). (TIF) [file pgen.1004464.s001.tif]

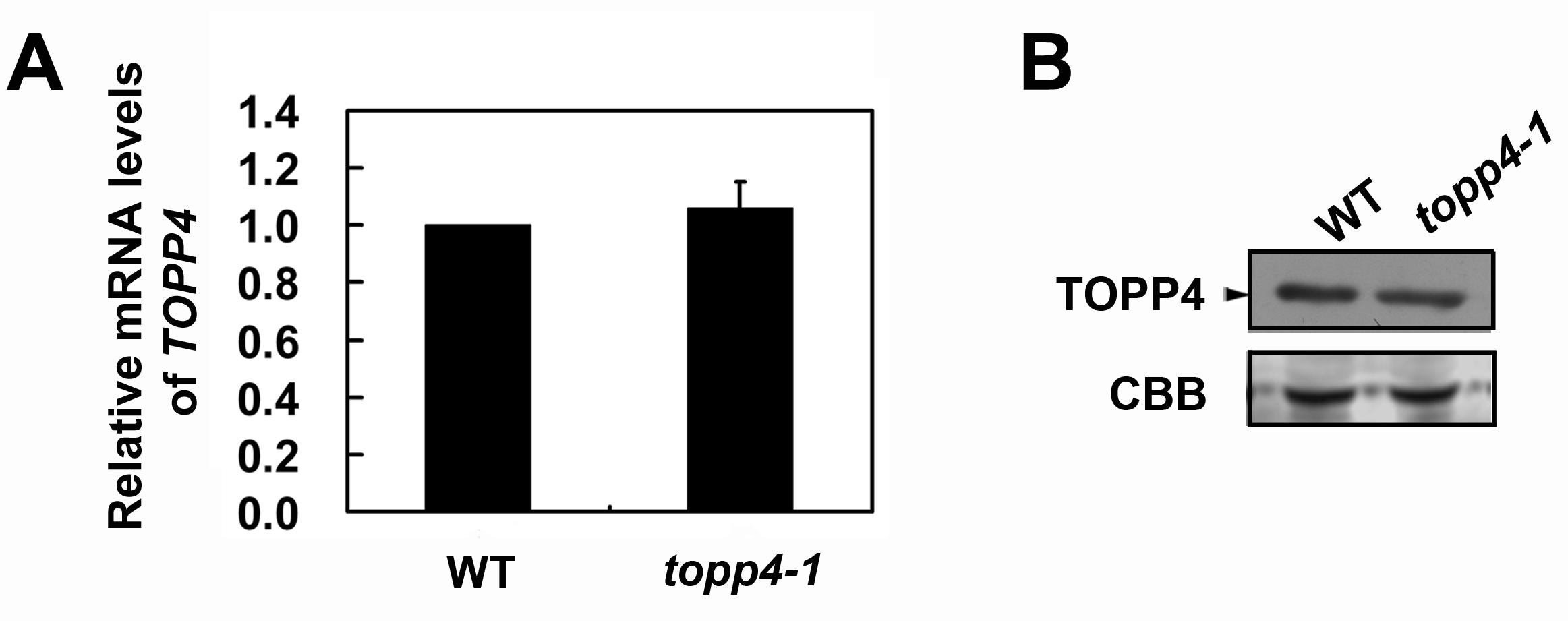

Supplement: Figure S2 — The expression levels of TOPP4 and the protein levels of TOPP4 in wild-type plant and the topp4-1 mutant. (A) Analysis of the TOPP4 expression in wild-type and topp4-1 seedlings by qRT-PCR. The expression level of wild type was set to 1.0. Error bar represents SE (n = 3). (B) The protein levels of TOPP4 in wild-type and topp4-1 plants determined by immunoblotting using antibody against TOPP4. (TIF) [file pgen.1004464.s002.tif]

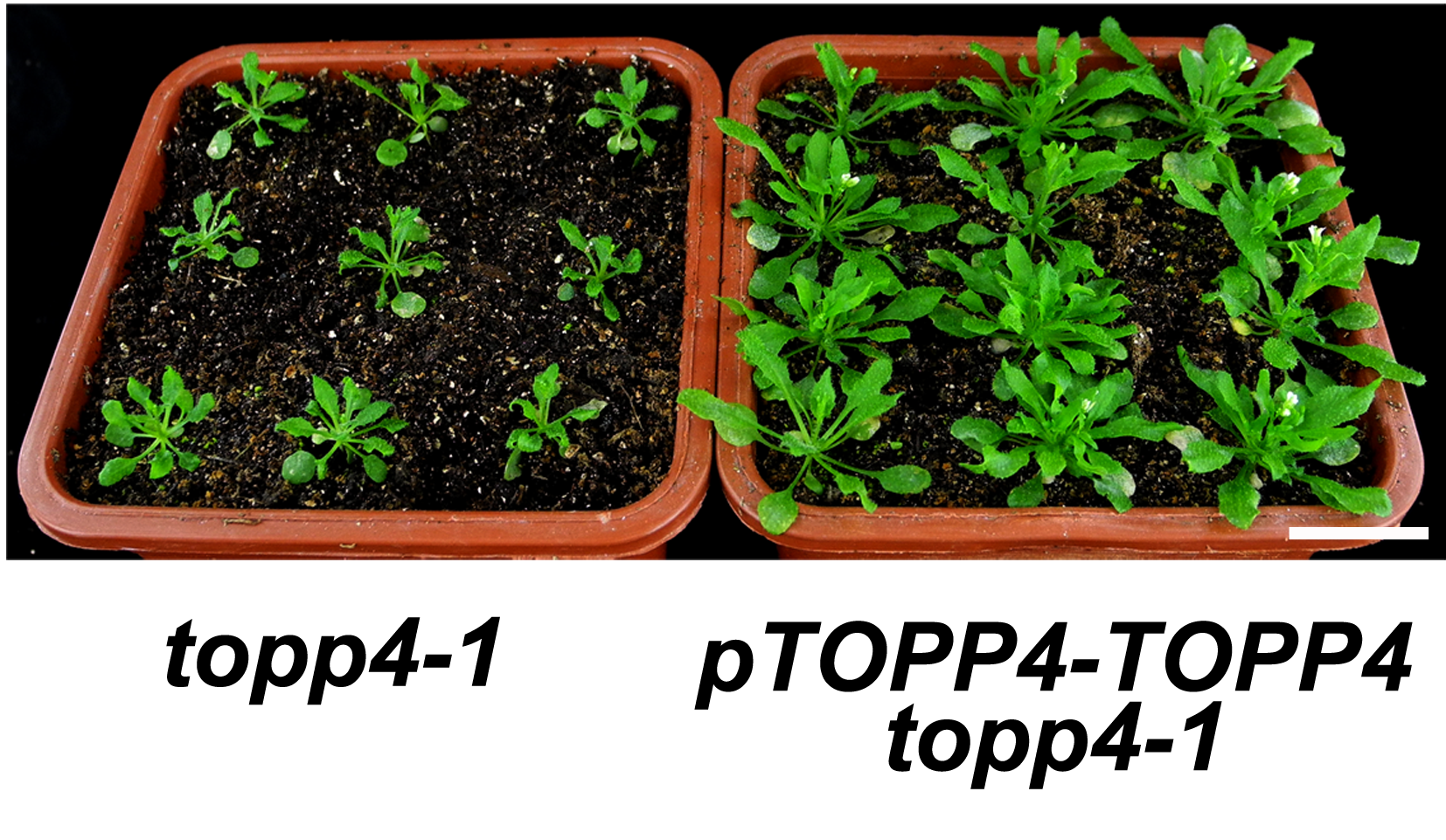

Supplement: Figure S3 — Transformed pTOPP4-TOPP4 into topp4-1 partially rescued the size of rosette leaves but did not rescue the dwarfed phenotype. Four-week-old topp4-1 and pTOPP4-TOPP4 topp4-1 transgenic plants are shown. Scale bar = 1 cm. (TIF) [file pgen.1004464.s003.tif]

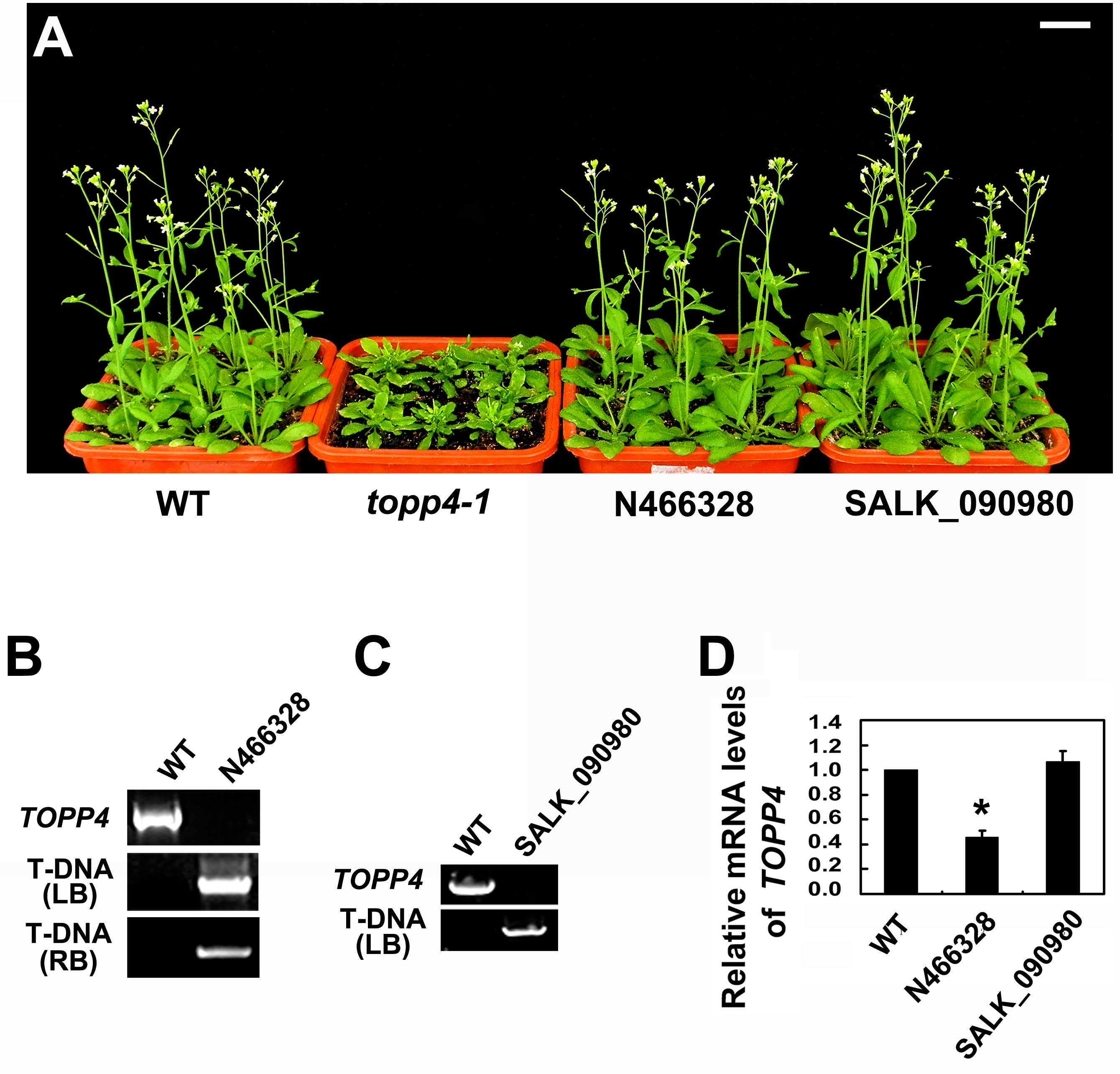

Supplement: Figure S4 — Two T-DNA insertion alleles of TOPP4 were identified from GABI-Kat and SALK T-DNA insertion databases. (A) Five-week-old wild-type, topp4-1, N466328, and SALK_090980 plants. (B) and (C) PCR-based analysis was used to identify the homozygous plants of N466328 (B) and SALK_090980 (C). (D) Analysis of the TOPP4 expression in wild-type, N466328, and SALK_090980 plants by qRT-PCR. The expression level of wild type was set to 1.0. Asterisk represents statistic differences based on Student's t test with P<0.05. Error bars represent SE (n = 3). Scale bar = 1 cm. (TIF) [file pgen.1004464.s004.tif]

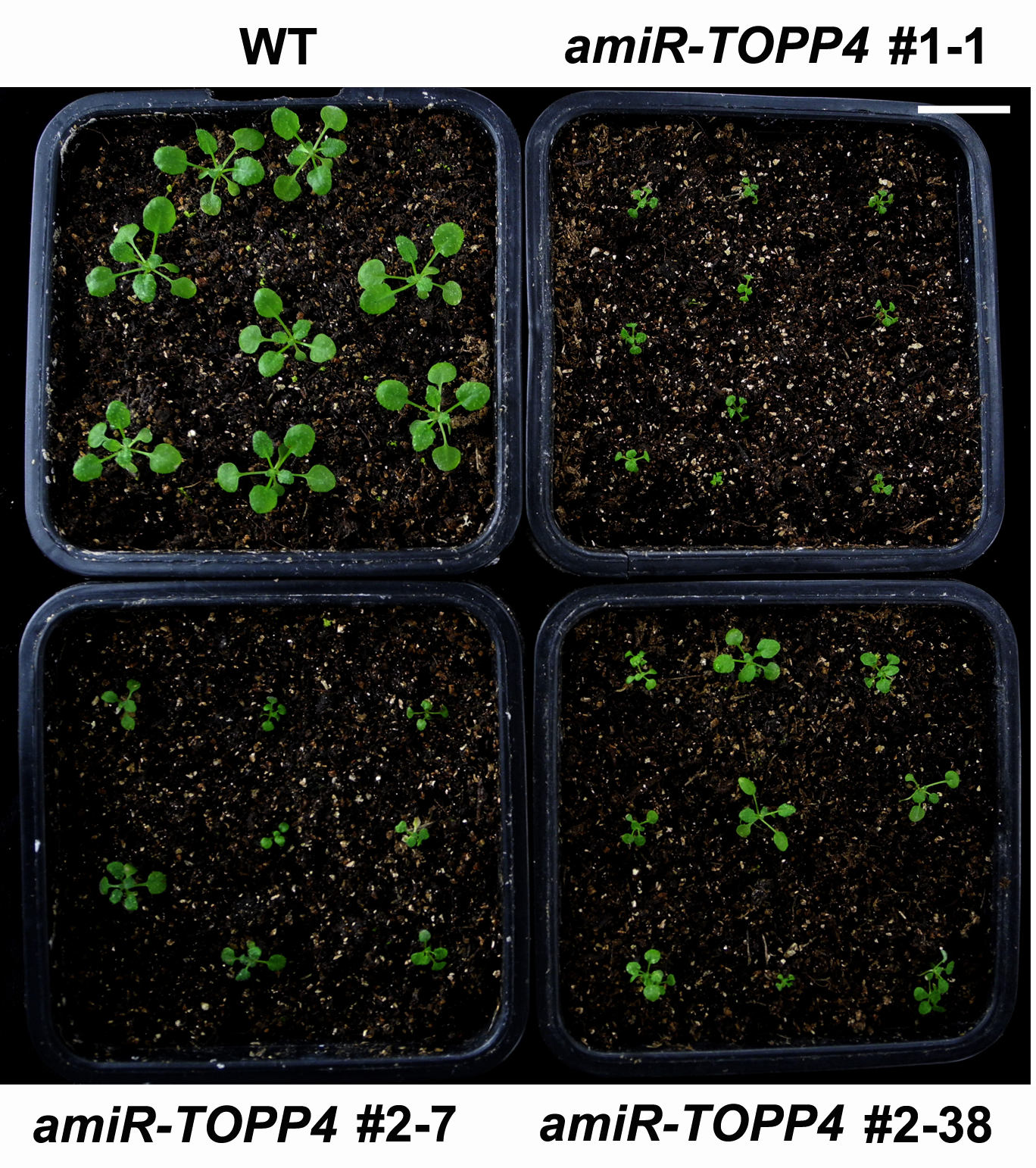

Supplement: Figure S5 — Phenotype of 2-week-old three amiR-TOPP4 transgenic lines. Scale bar = 1 cm. (TIF) [file pgen.1004464.s005.tif]

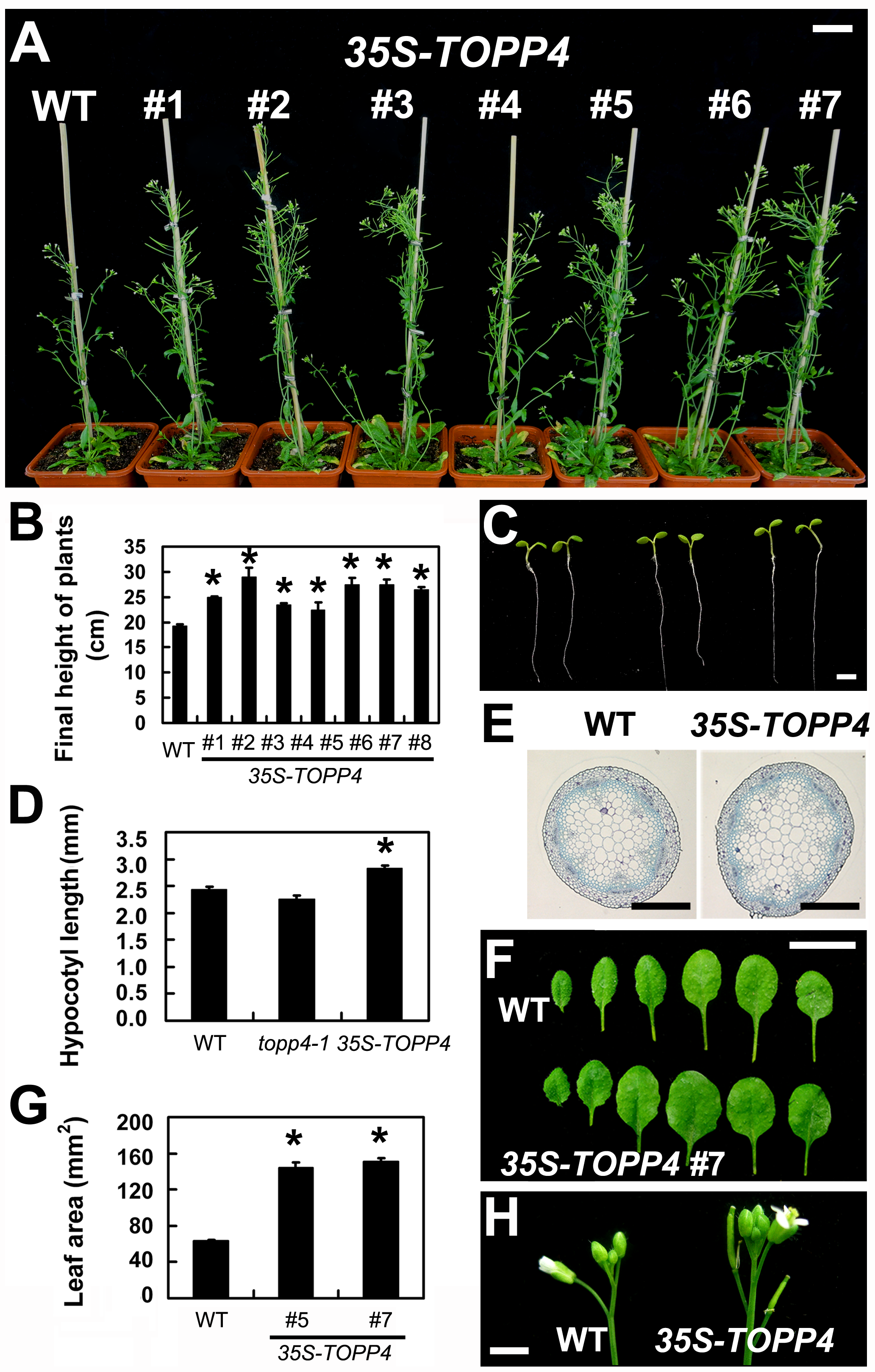

Supplement: Figure S6 — Overexpression of TOPP4 in wild-type plants increased inflorescence height and enhanced organ size. (A) Representative 7-week-old 35S-TOPP4 transgenic lines. (B) Final height of 7-week-old 35S-TOPP4 transgenic lines. Error bars represent SE (n = 20). (C) Ten-day-old wild-type, topp4-1, and 35S-TOPP4 seedlings (from left to right). (D) The hypocotyl length of 10-day-old wild-type, topp4-1, and 35S-TOPP4 plants. Error bars represent SE (n = 30). (E) Cross section of inflorescences of wild-type and 35S-TOPP4 plants. (F) Rosette leaves from wild-type and 35S-TOPP4 plants. (G) The area of the second pair of rosette leaves of wild-type and 35S-TOPP4 plants. Error bars represent SE (n = 30). (H) The top of inflorescences of wild-type and 35S-TOPP4 plants. Asterisks in (B), (D) and (G) represent statistic differences based on Student's t test with P<0.05. Scale bars = 2 cm in (A); 1 mm in (C) and (E); 1 cm in (F); 3 mm in (H). (TIF) [file pgen.1004464.s006.tif]

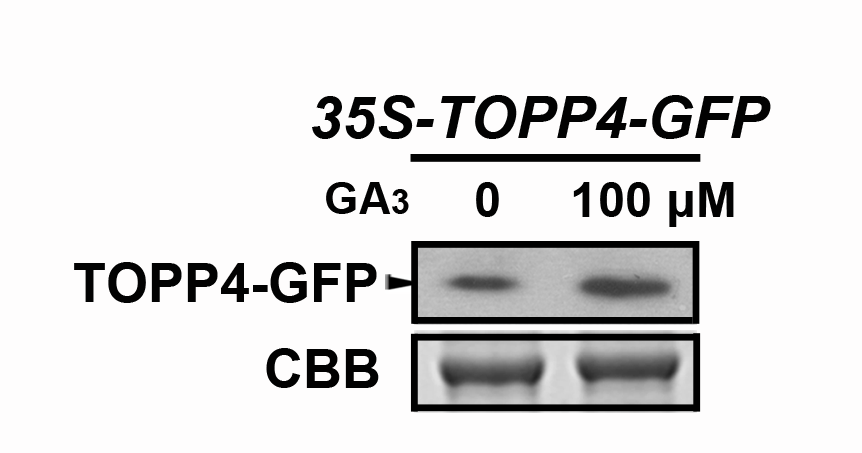

Supplement: Figure S7 — GA enhanced the TOPP4 protein level. The protein levels of TOPP4-GFP in 35S-TOPP4-GFP seedlings treated with or without 100 µM GA3. (TIF) [file pgen.1004464.s007.tif]

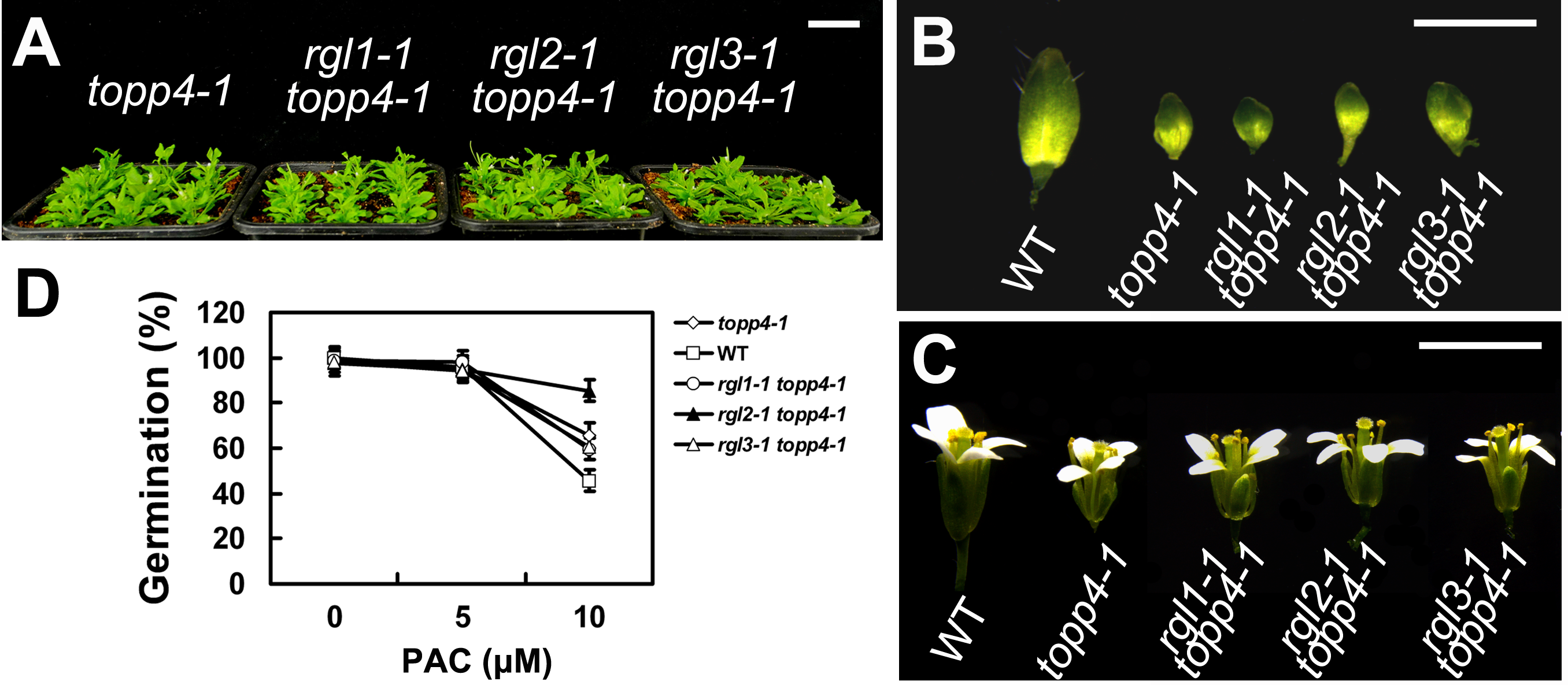

Supplement: Figure S8 — TOPP4 has no genetic interaction with RGL1, RGL2, and RGL3. (A) Representative 6-week-old topp4-1, rgl1-1 topp4-1, rgl2-1 topp4-1, and rgl3-1 topp4-1 plants. Scale bar = 1 cm. (B) and (C) Floral buds (B) and flowers (C) of topp4-1, rgl1-1 topp4-1, rgl2-1 topp4-1, and rgl3-1 topp4-1 plants. Scale bars = 0.5 cm. (D) Seed germination of topp4-1, wild-type, rgl1-1 topp4-1, rgl2-1 topp4-1, and rgl3 topp4-1 treated with different concentrations of PAC. Error bars represent SE (n = 30). (TIF) [file pgen.1004464.s008.tif]

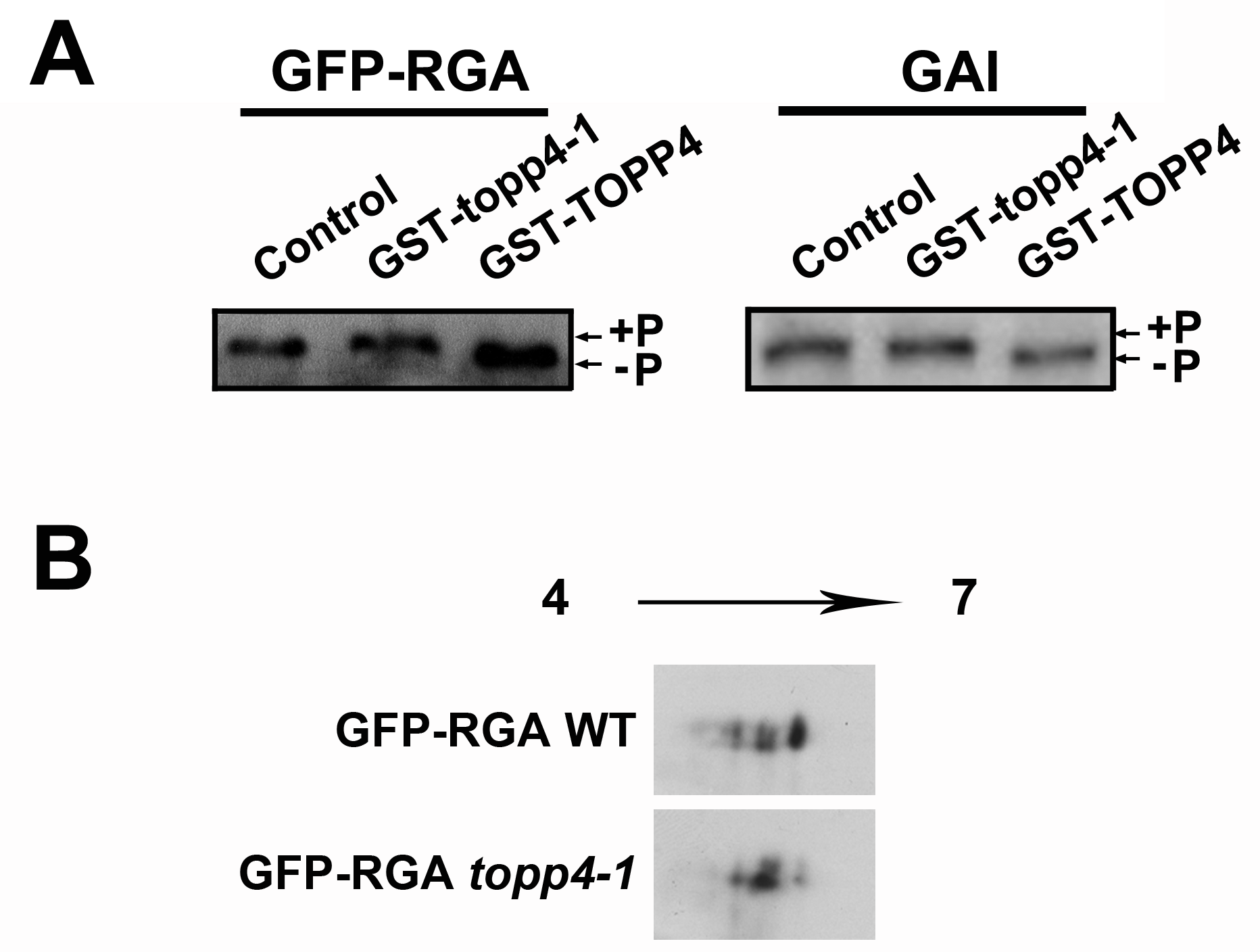

Supplement: Figure S9 — Dephosphorylation assays of RGA and GAI. (A) Immunoblotting assay of GFP-RGA and GAI proteins incubated with GST-topp4-1 or GST-TOPP4 from E. coli. Phosphorylated status, +P; dephosphorylated status, −P. (B) 2-DE analysis of posttranslational modification of GFP-RGA in wild-type and topp4-1 plants. (TIF) [file pgen.1004464.s009.tif]

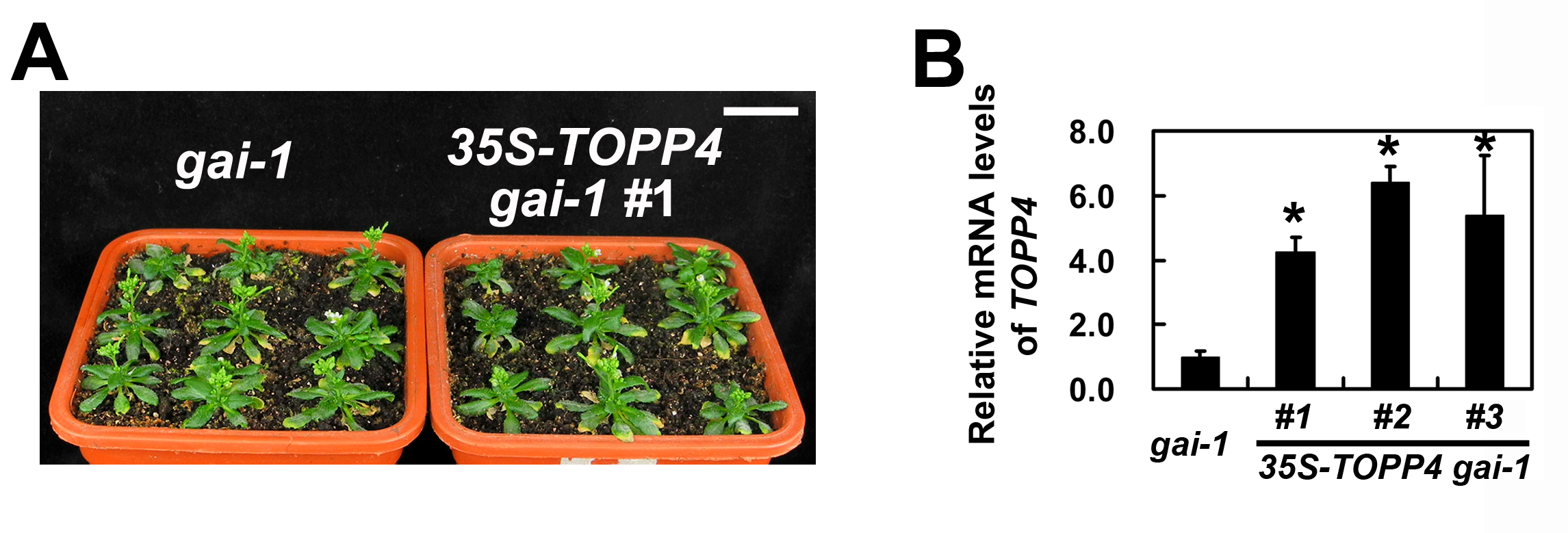

Supplement: Figure S10 — Overexpression of TOPP4 could not rescue the dwarfed phenotype of gai-1. (A) Representative 6-week-old gai-1 and 35S-TOPP4 gai-1 #1 plants. Scale bar = 1 cm. (B) Analysis of the TOPP4 expression in three representative 35S-TOPP4 gai-1 T2 transgenic lines by qRT-PCR. The expression level of gai-1 was set to 1.0. Asterisks represent statistic differences based on Student's t test with P<0.05. Error bars represent SE (n = 3). (TIF) [file pgen.1004464.s010.tif]

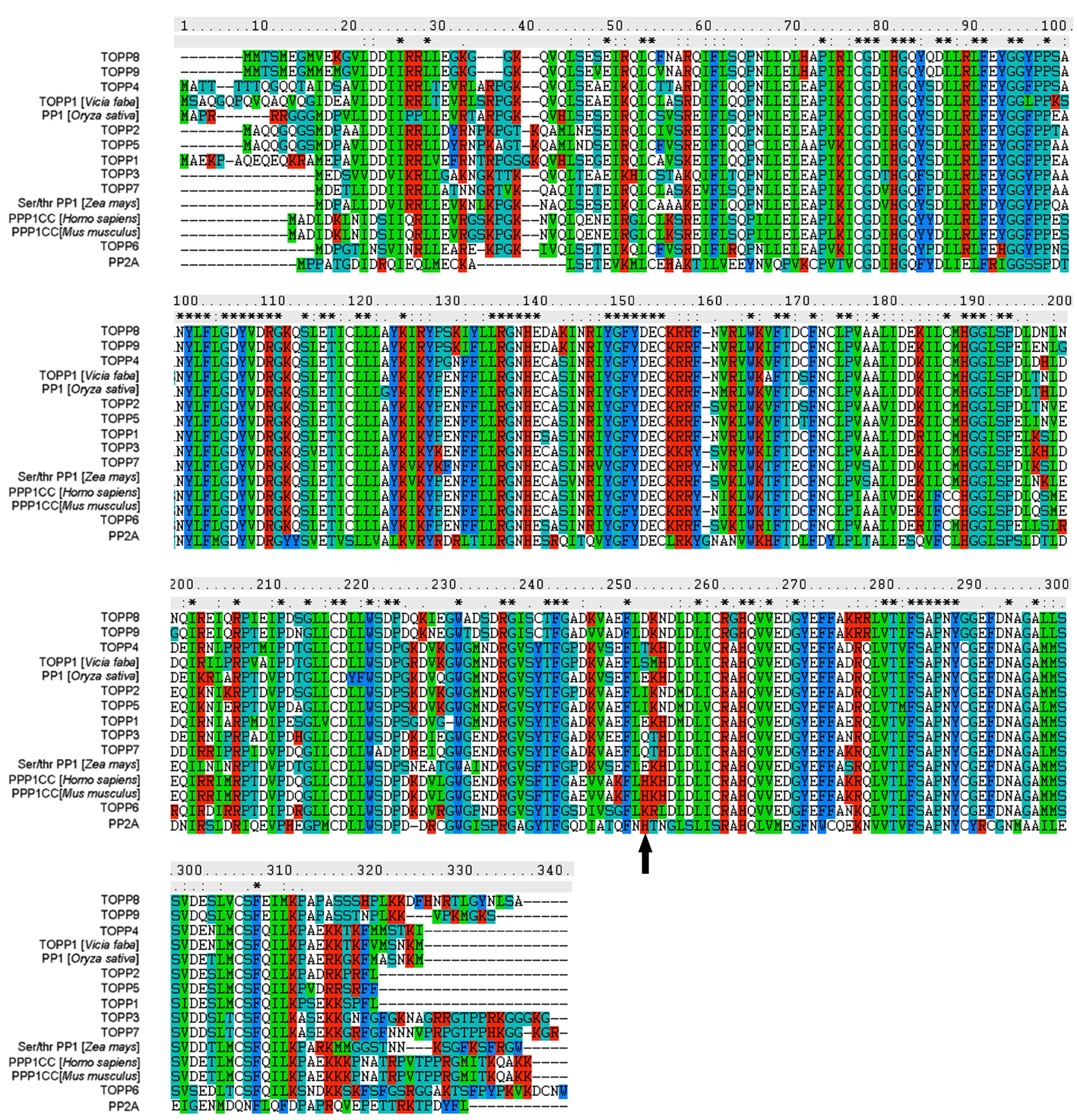

Supplement: Figure S11 — Amino acid sequence comparison among TOPP4, other TOPPs, and the PP1s of Oryza sative, Vicia faba, Zea mays, Homo sapiens, and Mus musculus showing high similarity to TOPP4. The numbers at the top of the sequences indicate the position of amino acid residues in corresponding proteins. Amino acids shared by all members are shaded in the same color and marked by asterisks. Thr246 in TOPP4 is indicated by arrow. (TIF) [file pgen.1004464.s011.tif]

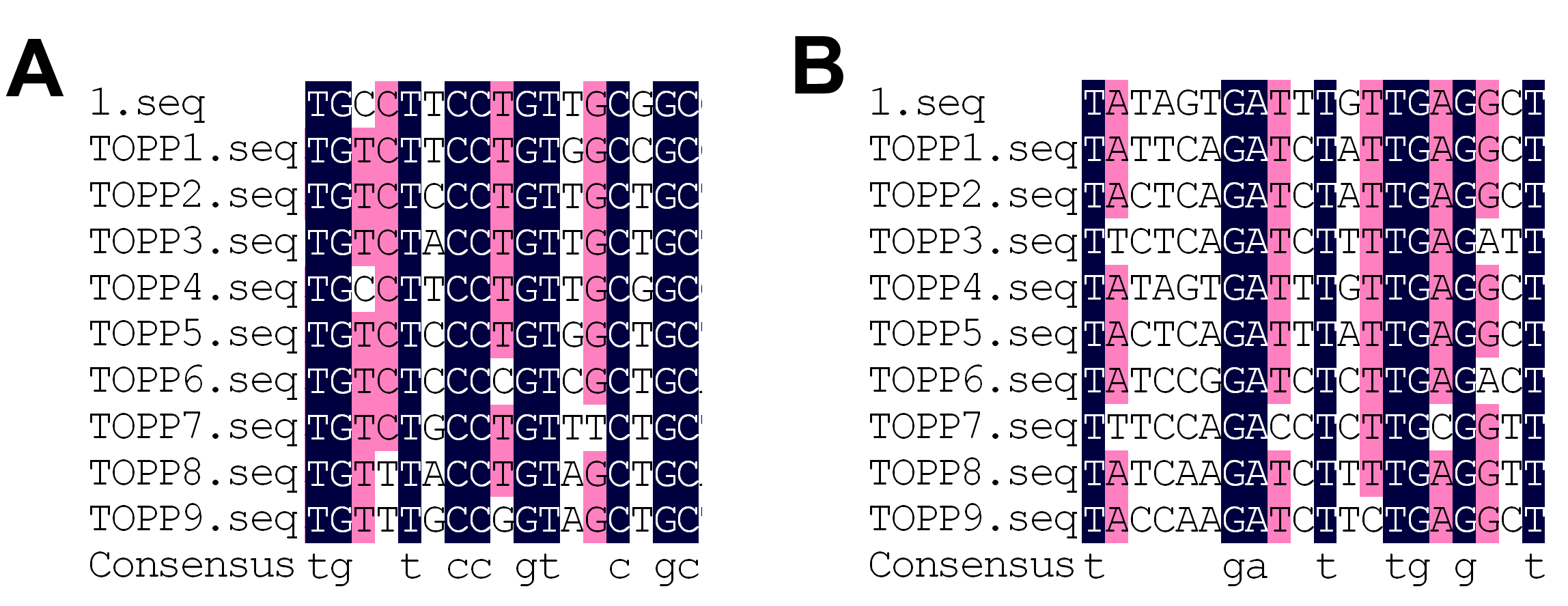

Supplement: Figure S12 — The alignment of the nucleotide sequence for targets of amiR-TOPP4 with the same regions of other TOPPs in Arabidopsis. (A) amiR-TOPP4-1. (B) amiR-TOPP4-2. (TIF) [file pgen.1004464.s012.tif]
