# Supplementary material for: Arabidopsis DELLA Protein Degradation Is Controlled by a Type-One Protein Phosphatase, TOPP4
Source: PLoS Genet. 2014 Jul 10;10(7):e1004464. doi: 10.1371/journal.pgen.1004464 (PMC4091783; doi:10.1371/journal.pgen.1004464)
Supplement: Table S1 — New developed In/Del and CAPS markers used for fine mapping in the topp4-1 mutant. (DOC) [file pgen.1004464.s013.doc]

**Table S1. New Developed In/Del and CAPS Markers Used for Fine Mapping in the *topp4-1* Mutant.**

| Name | Primer 1 (5’→3’) | Enzyme | Length of product (bp)  Col Ler |
| --- | --- | --- | --- |
| T3K9-78646  T11A7-33794  F27I1-17157  T28M21-91855  T5I7-29008  F12L6-66859  T16B24-61552  T28M21-47168 | GGATATTGGTGTTGACGAACTTCC  GCTCGTTTCTTCCTCCTCGTCG  ACGGTCCATATTCGAATTGGAG  GGCTCGGTTTATTCTTTGAGCAG  CCAGAAAGTGAATTTACCTAACC  CGGTTGGATAGTTTAGTTTTTCC  GCTCAGTCTCCATCTCAGACGC  CAAGAGTTTATGTCTGCGTCTGG  GCGCAAAACGTCGGTGAGTC  CACCATCAAACGCGGTGACCC  GTAACTGCAAACGCGTTGAC  GTCAACATGTATAGTGTCGT  CCGATTGATTCTATCGGAGG  GTTTCTTGCTCTGGACTCGG  ATATTCACAAAACATAGGGTACTGC  TGTGCTGTTTTGGTTTTCTT | -  -  -  -  -  Fok I  TaqI  TspE I | 265 171  315 299  214 210  220 210  238 234  86 157; 243  108 130; 238  200 ＜200；200＞200 |
