# Supplementary material for: Arabidopsis DELLA Protein Degradation Is Controlled by a Type-One Protein Phosphatase, TOPP4
Source: PLoS Genet. 2014 Jul 10;10(7):e1004464. doi: 10.1371/journal.pgen.1004464 (PMC4091783; doi:10.1371/journal.pgen.1004464)
Supplement: Table S2 — Primers used for genotype identification and qRT-PCR. (DOC) [file pgen.1004464.s014.doc]

**Table S2.** Primers Used for Genotype Identification and qRT-PCR.

| Primer name | Primer sequence |
| --- | --- |
| SALK_090980-F  SALK_090980-R  SALK_090980-LB  N466328-F  N466328-R  N466328-LB  N466328-RB  GAPC-F  GAPC-R  TOPP4-F  TOPP4-R  EXP8-F  EXP8-R  PRE1-F  PRE1-R | 5’-ACCCACAGGTAGTCTCGGAACAATCA-3’  5’-CGAATCAAATTGGGAAGATACCACAGA-3’  5’-CAGGATTTTCGCCTGCTGGGGC-3’  5’-CTTCTCTGTGATTTGCTCTGGT-3’  5’-CTTGTTGTCCAATTTAGATGCTC-3’  5’-ATATTGACCATCATACTCATTGC-3’  5’-GTGGATTGATGTGATATCTCC-3’  5’-ACCACACGGGAACTGTAACC-3’  5’-GGCTATCAAGGAGGAATCCG-3’  5’-ACTCTGTTTGCCTCTGTCCA-3’  5’-AATCTGTGGTGACATACATGG-3’  5’-CATGTATGAAGAAAGGAGGAATAAG-3’  5’-AACTGCCAATTAGAAGGAGCCACG-3’  5’-CAGCCTCGAAAGTATTGCAAG-3’  5’-TTCTAATAACGGCGGCTTCAG-3’ |
